# Ethyl‐*N*‐dodecanoyl‐l‐arginate hydrochloride combats pathogens with low‐resistance generation by membrane attack and modifies gut microbiota structure

**DOI:** 10.1111/1751-7915.13514

**Published:** 2019-11-22

**Authors:** Ting Shao, Tingting Fan, Wenshu Tang, Yanting Sun, Song Gao, Huang Chen, Zhenliang Sun, Mingyao Liu, Zhengfang Yi

**Affiliations:** ^1^ East China Normal University and Shanghai Fengxian District Central Hospital Joint Center for Translational Medicine Shanghai Key Laboratory of Regulatory Biology, Institute of Biomedical Sciences and School of Life Sciences East China Normal University 200241 Shanghai China; ^2^ Department of Anal and Intestinal Diseases Longhua Hospital Shanghai University of Traditional Chinese Medicine 200032 Shanghai China; ^3^ Shanghai University of Medicine and Health Sciences Affiliated with Sixth People’s Hospital South Campus Shanghai 201499 China

## Abstract

Ethyl‐*N*‐dodecanoyl‐l‐arginate hydrochloride (LAE, ethyl lauroyl arginate HCl) is a cationic surfactant used as a food preservative with broad‐spectrum antibacterial activities. However, its resistance development, influences on gut microbiome and molecular target are unclear. In this study, bacteria were stimulated by LAE for 30 days to test the bacterial resistance. Several infected animal models were used to evaluate the antibacterial effect of LAE *in vivo*. Mice were orally treated with LAE to test its effect on animal growth. The influence of LAE on mice gut microbiome was analysed by 16S rDNA sequencing. The results indicated that *Escherichia coli* did not develop resistance to LAE. LAE significantly combats bacterial infection in mice, ducklings and piglets. Moreover, LAE promotes mouse weight gain without changing body composition or reducing animal vitality, and induces lower hepatotoxicity than ampicillin. In the mouse gut microbiome assessment and characterization, LAE modifies host gut microbiota structure. Mechanistically, LAE specifically binds to acidic phospholipids including phosphatidylserine, depolarizes the membrane and disrupts the bacterial membrane followed by bacterial growth inhibition. This study investigates the molecular mechanism of LAE as well as its antibacterial functions in poultry and livestock. Our data suggest LAE is a potential antibacterial agent in animal health.

## Graphical abstract

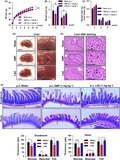

## Introduction

Resistance development limits the useful lifespan of antibacterial agents, and the consequent failure of antibiotic therapy has restored infectious diseases to the list of leading causes of death worldwide, causing a public health crisis (2018). Antibiotics fight against bacterial infection, and they are also used as growth promoters in animals to increase weight gain partly by attenuation of the intestine wall (Hendrickx, *et al.*, 2015), such as ampicillin (AMP), a β‐lactam antibiotic with a broad spectrum (Butaye, *et al.*, 2003; Cho, *et al.*, 2012; Danzeisen, *et al.*, 2015; Redondo, *et al.*, 2015). However, approximately 75% of antibiotics have poor metabolic activity and are excreted into the environment or stay in the animal tissue (Chee‐Sanford, *et al.*, 2009). Diverse, abundant and potentially mobile antibiotic resistance genes in farm samples suggest that intensive animal husbandry is believed to be a major contributor to the increased environmental burden of antibiotic resistance (Shi, *et al.*, 2011; Zhu, *et al.*, 2013; Huang, *et al.*, 2015).

Antibacterial drugs that inhibit cell wall synthesis, DNA replication and protein synthesis need to enter bacterial cells, thus giving bacteria an opportunity to develop tolerance by preventing drugs entry, increasing drugs efflux, changing drug targets and degrading drugs (Kupferschmidt, 2016). Charged molecules interact with microbial membranes through electrostatic interactions, thus depolarizing the membrane and physically damaging the bacterial morphology (Zasloff, 2002). As membrane targeting antibacterial molecules destroy the first line of bacteria defence, they render bacteria less likely to develop resistance (Wenzel, *et al.*, 2014; Lam *et al.*, 2016). Compared with eukaryotic cells, prokaryotic cell membranes contain more negatively charged acid phospholipids such as phosphatidylserine (Zasloff, 2002; Lam *et al.*, 2016). In addition, the acid phospholipids were evenly distributed on the internal and external phospholipid bilayers of prokaryotic cells, while it was only distributed on the internal phospholipid bilayer of eukaryotic cells (Matsuzaki, 1999). Acidic phospholipids may be the bacterial target of non‐toxic cationic antibacterial compounds.

In addition to inducing antibiotic‐resistant bacteria (Levy and Marshall, 2004; Gullberg, *et al.*, 2011; Wang, *et al.*, 2017; Zurfluh, *et al.*, 2017), antibiotics also disturb the gut microbiome because they are unfavourable to the growth of probiotic bacteria and promote the expansion of pathogens (Pallav, *et al.*, 2014; de Gunzburg, *et al.*, 2018). Probiotic bacteria are the bacteria that confer health benefits to the host, including increased body weight and improved immunity (Turroni, *et al.*, 2014). *Bifidobacterium* and *Lactobacillus* are the most widely studied probiotic bacteria in the human or animal gut possessing distinct beneficial attributes by strengthening the intestinal barrier, modulating the immune response and antagonizing pathogens either by the production of antimicrobial compounds or through competition for mucosal binding sites (Gao, *et al.*, 2017). However, high levels of probiotic bacteria are only found in infants and newborn animal guts (Turroni, *et al.*, 2010). Hence, probiotics are used in feeding to get effects on absorption and utilization of feed, increasing the productivity of various animals (Casey, *et al.*, 2007; Samli, *et al.*, 2007). The use of antibiotics as feed additives has been restricted in several countries due to their side‐effects (Gadde, *et al.*, 2017), so it is urgent to develop safer candidates.

Ethyl‐*N*‐dodecanoyl‐l‐arginate hydrochloride (LAE, CAS: 60372‐77‐2) is a food‐grade cationic surfactant that has strong antimicrobial activities against a wide range of microorganisms, including bacteria, fungi and yeast, with low animal toxicity (Rodriguez, *et al.*, 2004; Ruckman, *et al.*, 2004; Hawkins, *et al.*, 2009). LAE has been considered as a safe food additive by the Food and Drug Administration (FDA) since 2005 and the European Food Safety Agency (EFSA) since 2007 (Muriel‐Galet, *et al.*, 2012). Existing studies only show its ability to destroy bacterial cell membranes (Rodriguez, *et al.*, 2004). A previous study (Schwartz, *et al.*, 1997) on the mammalian metabolism of LAE in rats indicated an almost complete absorption of LAE, and it was completely metabolized to endogenous components, including CO_2_, water, ornithine, urea and l‐arginine (Hawkins, *et al.*, 2009). However, its molecular target, effects on drug resistance development and influence on antibacterial activity and the gut microbiome *in vivo* are not clarified.

In this study, we describe the evaluation of LAE as an antimicrobial veterinary drug and growth promoter. 16S ribosomal DNA (rDNA) amplicon sequencing was performed to analyse the influence of LAE on mouse gut microflora. Moreover, its antibacterial mechanism was also preliminarily investigated.

## Results

### Bacterial resistance to LAE is much lower than to antibiotics

**Figure 1:**
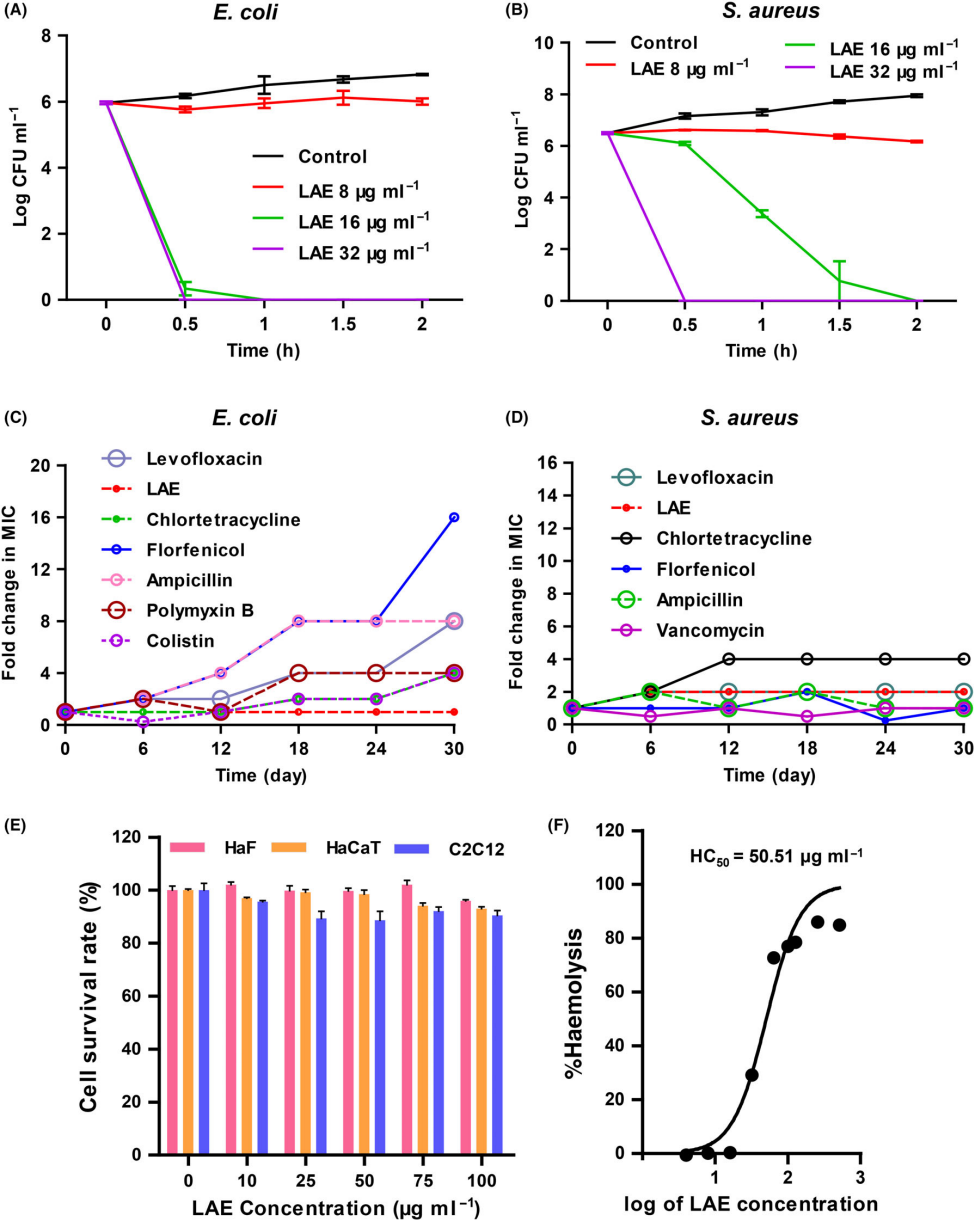
Lower acquisition of resistance in the presence of sub‐MIC levels of LAE compared with antibiotics. A. Viability of exponential‐phase Gram‐negative bacteria (ATCC 25922) *E. coli* or (B) Gram‐positive bacteria (ATCC 29213) *S. aureus* treated with LAE at 8 μg ml^−1^, 16 μg ml^−1^ and 32 μg ml^−1^ (*n* = 3). C. *E. coli* or (D) *S. aureus* were serially passaged in MHB containing sub‐MIC levels of LAE or indicated antibiotics. The change in the MIC of drugs against both strains is shown on the y‐axis over 30 days (450 generations of bacterial growth). E. The cell survival rate of HaF, HaCaT and C2C12 cells treated with LAE at the indicated concentrations for 48 h (*n* = 3). F. The haemolysis rate of rat blood cells treated with LAE at 4‐512 μg ml^−1^ for 1 h (*n* = 3).

We evaluated the antibacterial efficacy of LAE by determining its minimum inhibitory concentration (MIC) and minimum bactericidal concentration (MBC) against *Staphylococcus aureus* and *E. coli*. The MIC of LAE against *E. coli* and *S. aureus* was 16 and 8 μg ml^−1^ respectively. The MBC of LAE against *E. coli* and *S. aureus* was both 16 μg ml^−1^ (Table 1). According to the time‐kill assays, it took less than 30 min for LAE to reduce *E. coli* and *S. aureus* population from 9.33 × 10^5^ CFU ml^−1^ and 3.13 × 10^6^ CFU ml^−1^, respectively, to zero at 32 μg ml^−1^ (Fig. 1 A,B). Many antibiotics have become ineffective soon after introduction due to bacterial resistance, so the ability of drugs to induce bacterial resistance has been tested *in vitro*. There was no change in the MIC of LAE against *E. coli* after 30 days of stimulation at sub‐MIC concentration, which remained 16 μg ml^−1^ from beginning to end. Under the same experimental conditions, the MIC of several antibiotics against *E. coli* increased to varying degrees (Fig. 1C). For *S. aureus*, the MIC of chlortetracycline increased to 4 times the initial MIC, and the MIC of LAE and levofloxacin increased to 2 times the initial MIC. The MIC of florfenicol, ampicillin and vancomycin remained unchanged (Fig. 1D). This study suggested that LAE induced lower drug resistance than antibiotics tested in this research.

**Table 1:** *In vitro* antibacterial activity of LAE.

Drugs		MIC^a^ (μg ml^−1^)		MBC^b^ (μg ml^−1^)	
		*E. coli* (ATCC 25922）	*S. aureus* (ATCC 29213）	*E. coli* (ATCC 25922）	*S. aureus* (ATCC 29213）
LAE		16	8	16	16
β‐Lactams	Ampicillin	0.125	2048	8	>2048
	Cefazolin	2	2	16	16
Polypeptides	Polymyxin B	8	512	64	>2048
	Colistin	64	>2048	512	>2048
	Vancomycin	1	2	4	16
Quinolones	Levofloxacin	1	0.25	8	0.5
Chloramphenicols	Chloramphenicol	4	4	64	32
	Florfenicol	2	8	32	32
Tetracyclines	Chlortetracycline	0.5	64	4	256

a MIC is defined as the minimum concentration of an antimicrobial agent at which no visible microbial growth is observed.

b MBC is defined as the minimum concentration of an antimicrobial agent at which no bacterial colony on agar plates is observed. Identical MIC values were obtained across three replicates completed in two independent experiments.

### LAE significantly combats bacterial infection in several species of mammals and poultry

**Figure 2:**
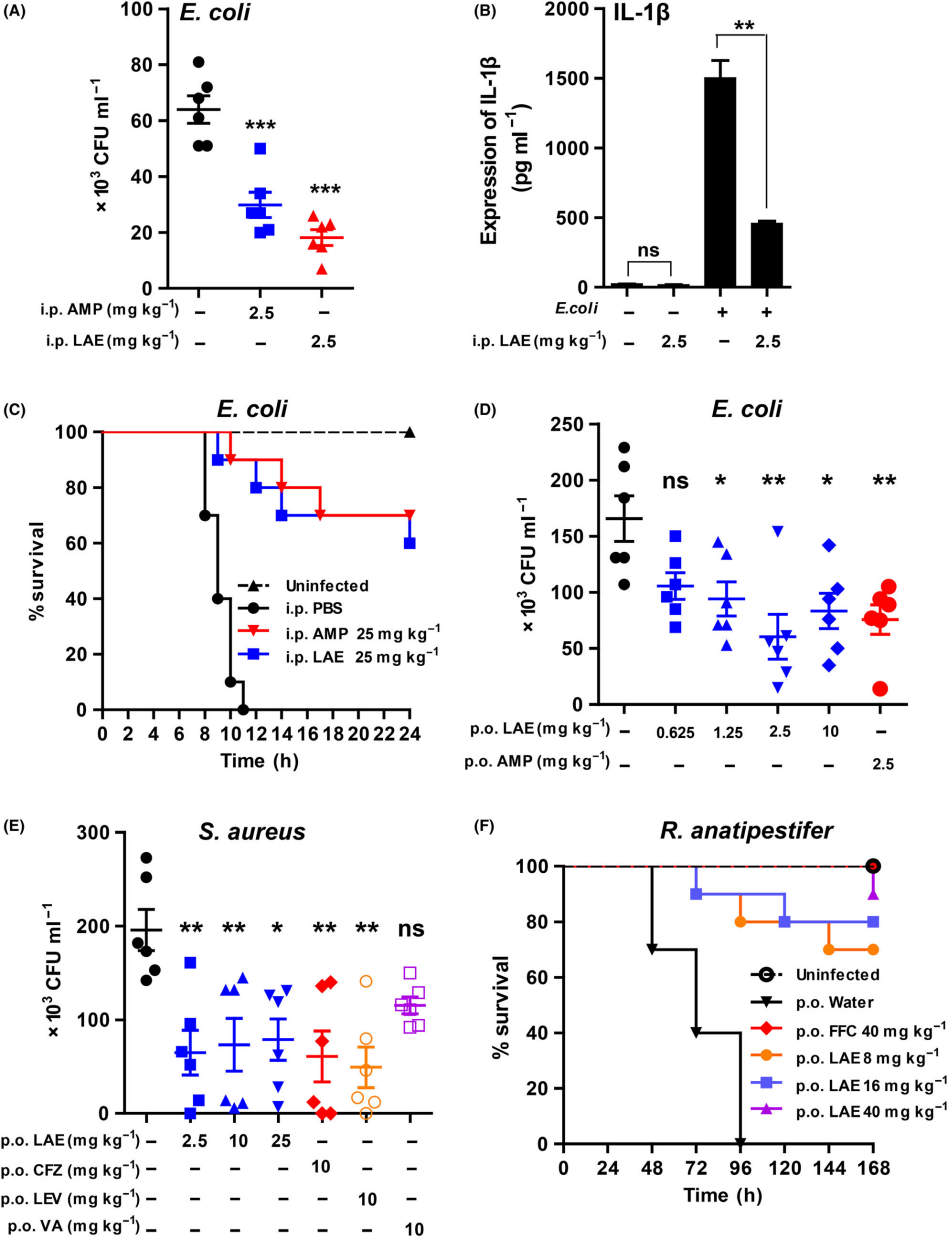
LAE combats bacterial infection in infected animals. A. Efficacy of intraperitoneal injection of LAE on the clearance of pathogens in *E. coli*‐infected mice (*n* = 6). Mice were challenged with 10^8^ CFU *E. coli* (*E. coli,* ATCC 25922) through intraperitoneal (i.p.) injection. An hour later, mice were divided randomly into three groups and treated with an intraperitoneal injection of PBS, LAE or antibiotic at the indicated doses. After 24 h, *E. coli* was obtained by abdominal cavity lavage and colony formation on TSA plates was quantified. AMP, ampicillin. B. Expression of IL‐1β in peritoneal fluid. C. Effect of LAE on survival rate of *E. coli‐*infected mice (*n* = 10). Three groups of BALB/c mice were divided and challenged with 10^9^ CFU *E. coli* 1 h before drug treatment, and the mice were checked every 2 h to obtain a survival curve. D. Efficacy of oral LAE on the clearance of pathogens in *E. coli‐*infected mice (*n* = 6). Mice were challenged with 10^8^ CFU *E. coli* through intraperitoneal (i.p.) injection. An hour later, mice were divided randomly into six groups and orally treated with PBS, LAE or antibiotics at indicated doses. After 24 h, *E. coli* was obtained by abdominal cavity lavage and colony formation on TSA plates was quantified. E. Efficacy of oral LAE on the clearance of pathogens in *S. aureus*‐infected mice (*n* = 6). BALB/c mice were infected with 10^8^ CFU *S. aureus.* An hour later, mice were divided randomly into seven groups and orally treated with PBS, LAE or antibiotics at indicated doses. After 24 h, *S. aureus* was obtained by abdominal cavity lavage and colony formation on TSA plates was quantified. CFZ, cefazolin, FFC, florfenicol; LEV, levofloxacin, VA, vancomycin. F. Effect of LAE on survival rate of *R. anatipestifer* (shown as *R. anatipestifer*)*‐*infected Cherry Valley ducklings (*n* = 10).

To validate the effect of LAE against pathogens *in vivo,* we set up three different infected animal models including mouse peritonitis model, piglets yellow scour and duckling *Riemerella anatipestifer* infection model. First, a mouse peritonitis model was set up by intraperitoneal injection of bacteria to monitor the amount of pathogens and mouse survival. As shown in Fig. 2A, the total quantity of pathogenic *E. coli* in the peritoneal cavity was significantly reduced in LAE intraperitoneal injected (i.p.) mice (*P* = 0.0004). LAE (i.p.) reduced the level of IL‐1β that increased in mice as a result of bacterial infection (Fig. 2B, *P* = 0.0052). Accordingly, the survival of LAE‐treated mice was also significantly extended when compared with that of control groups (Fig. 2C, *P* < 0.0001), suggesting a key role of LAE in the clearance of invading bacteria. LAE was also introduced through oral gavage and reduced the *E. coli* (Fig. 2D) and *S. aureus* (Fig. 2E) number *in vivo*. These results suggested that LAE reduced the number of Gram‐positive and Gram‐negative pathogens by intraperitoneal injection or oral intake in mammals.

The pig farm selected as the experimental site had a yellow scour breakout in Henan province, China. 118 three‐ to thirteen‐day‐old ill piglets were treated with 10 mg kg^‐1^, 50 mg kg^‐1^ LAE or 50 mg kg^‐1^ gentamicin three times per day for 4 days. The survival rate of 50 mg kg^‐1^ and 10 mg kg^‐1^ LAE group was 78.57% and 72.2% respectively. The survival rate of 50 mg kg^‐1^ gentamicin group was 77.5% (Table S1). In this experiment, LAE showed better effects on yellow scour than gentamicin at the same dose.

*Riemerella anatipestifer* is a Gram‐negative pathogen, which seriously harms duck farming. Ducklings were infected with *R. anatipestifer* by intramuscular injection 8 h after the first LAE oral treatment, followed by LAE treatment three times at 8‐h intervals. All ducklings without drug treatment died within 4 days (Fig. 2F, *P* < 0.0001), while treatment with 40 mg kg^‐1^ LAE kept all ducklings alive until day 6, with only one duckling dying on the 7th day. Even the lowest dose (8 mg kg^‐1^) resulted in a 70% survival rate on the last day of the experiment. In summary, LAE is effective in treating bacterial diseases in poultry and mammals.

### LAE promotes weight gain without changing body composition or reducing animal vitality and shows lower organ toxicity than antibiotic treatment

**Figure 3:**
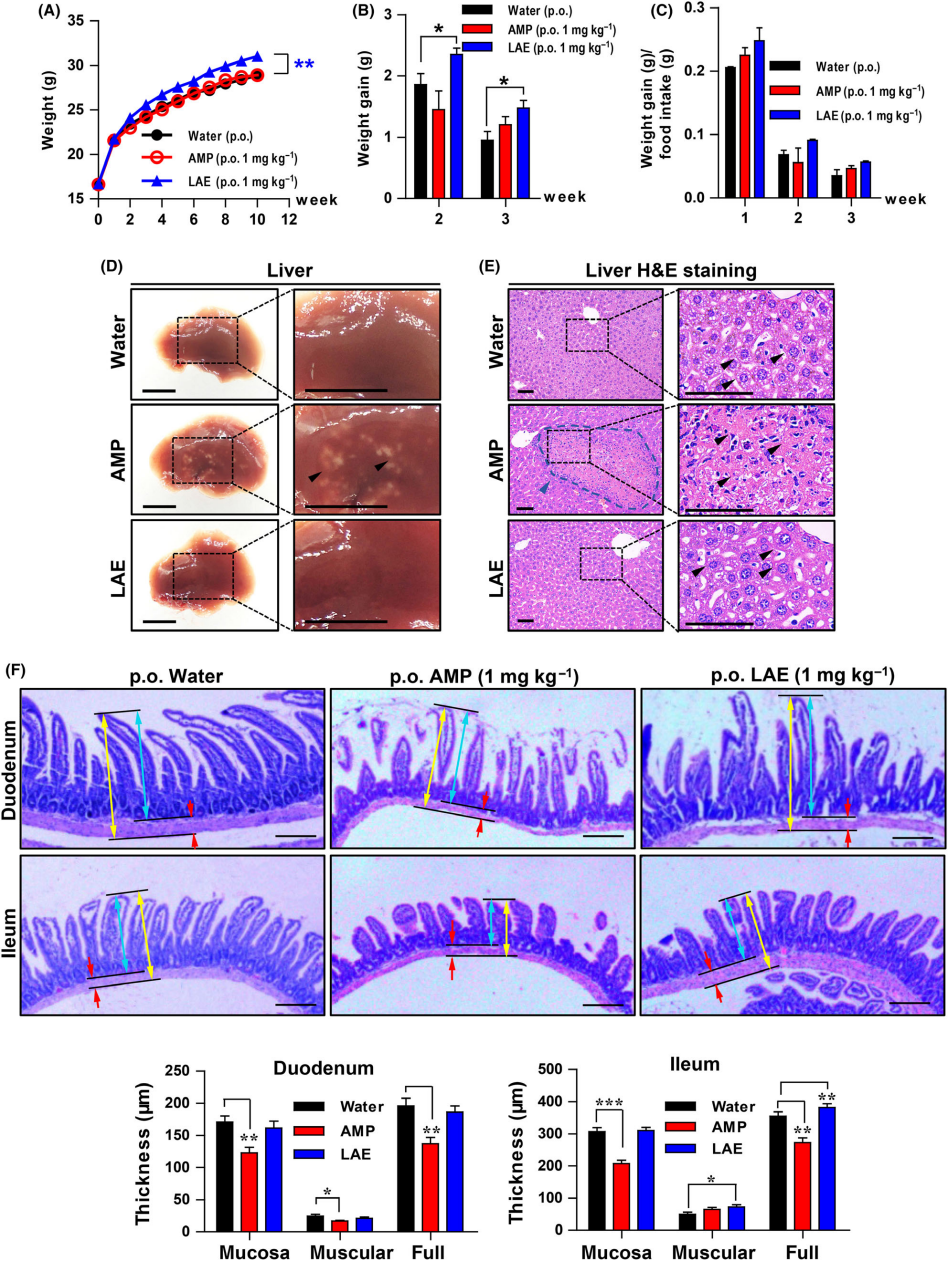
LAE promotes weight gain and shows less organ toxicity than ampicillin. 21‐day‐old male BALB/c mice were exposed to LAE or ampicillin (AMP), or untreated control (water) drinking water for 10 weeks (*n* = 10). A. LAE promoted growth. B. Weight gain was significantly increased in LAE group compared with water group. C. LAE enhances feed efficiency in the first, second and third weeks. The mouse livers were harvested, photographed (D, black arrows, disease area; scale bar, 1 cm), sectioned and stained with H&E (E). Scale bar, 100 μm; blue dotted line indicated by blue arrows, inflammatory cells; black arrows, normal hepatocyte with cell borders, while AMP group hepatocytes are aberrant. F. The mouse duodenum and ileum were sectioned and stained with H&E. Blue arrows, mucosa layer; red arrows, muscular layer; yellow arrows, full layer; scale bar, 200 μm. The graph indicates the mean thickness of mouse duodenum and ileum (*n* = 5).

To detect the effects of long‐term LAE intake on animals, we added LAE (1 mg kg^‐1^) or ampicillin (AMP, 1 mg kg^‐1^) to the drinking water of four‐week‐old male BALB/c mice for 10 weeks. In the second and third weeks, the LAE group had a significantly higher increase in body weight gain compared with the untreated control group, which was 26.61% (*P* = 0.0017) and 55.37% (*P* = 0.027) respectively (Fig. 3B). In the last week, 7.43% (*P* = 0.0011) body weight improvement in the LAE group was observed compared with the untreated water group (Fig. 3A). Feed utilization rate was also improved by LAE. To be specific, the ratio of weight gain/feed intake in the first, second and third weeks was increased by 20.5%, 30.32% and 61.37% respectively (Fig. 3C). The body fat ratio and lean ratio were not influenced by drug treatment (Fig. S1 A,B). The body weight improvement of the LAE group was not accompanied by a change in animal vitality or respiratory metabolism (Fig. S1 C, D, F, G). The AMP group did not show significant difference compared with the water group in the above results. Although there was no significant difference among three groups in the organ index (Fig. S1E), the organ photographs (Fig. 3D and Fig. S2) revealed several white non‐raised spots on the surface of the AMP group (3 of the 10) mouse livers (Fig. 3D, black arrows). Several disordered areas of cells surrounded by inflammatory cells (Fig, 3E, blue dotted area indicated by blue arrows) were observed by H&E staining, which indicated hepatotoxicity of ampicillin. An attenuation of the intestinal wall in the AMP treatment group was also observed by H&E staining (Fig. 3F). The thickness of the intestine wall in the AMP group was significantly decreased compared with controls. LAE increased the muscular and the full layer thickness of the ileum. Other organs of the LAE group did not show visible differences compared with the control water group, whether assessed by organ index, photograph or H&E staining. These results suggested that LAE promoted healthier animal growth than antibiotic (i.e. ampicillin) treatment, while ampicillin had potential hepatotoxicity.

### LAE modifies host gut microbiota structure

**Figure 4:**
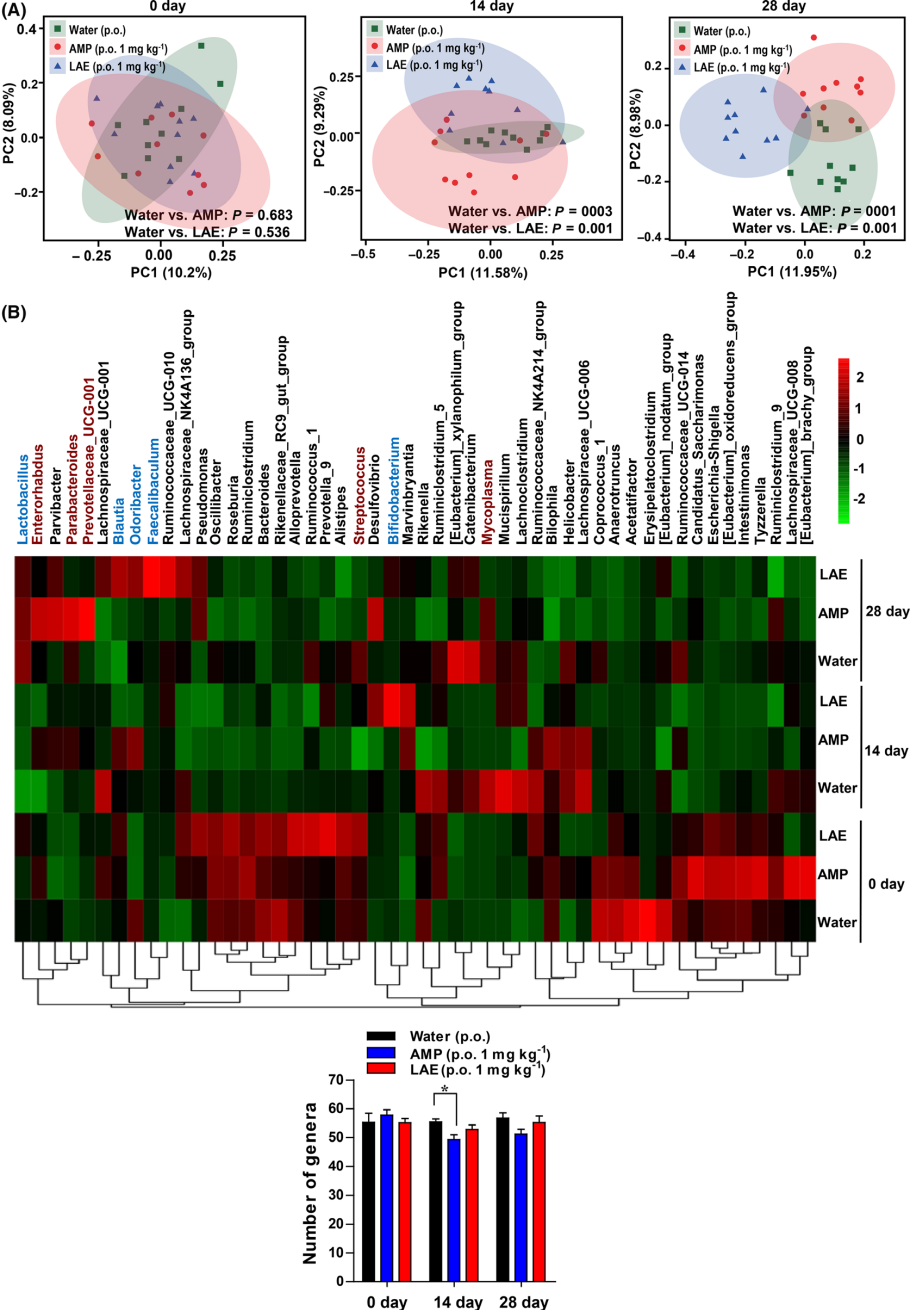
LAE modulates the gut microbiota structure. BALB/c mice were exposed to LAE (1 mg kg^‐1^) or ampicillin (AMP, 1 mg kg^‐1^) in drinking water or kept as untreated controls (water) for 10 weeks (*n* = 10 per group). A. Principle component analysis (PCA) of community composition at genus level on day 0/14/28. B. Heat map of relative abundance of top 50 bacteria at the genus taxonomic level (blue letters, probiotic microorganisms; red letters, disease‐related microorganisms). C. The number of microbial groups (*n* = 10) at the genus taxonomic level.

Antibacterial drugs usually have negative effects on the gut microbiome, such as reducing probiotic abundance and microbial diversity (Kofteridis, *et al.*, 2009; Pallav, *et al.*, 2014). Principal component analysis (PCA) of the community composition at the genus level on the day before drug administration and on the 14th and 28th day of drug treatment showed that both LAE and AMP altered the gut microbial community structure significantly (Fig. 4A). *Bifidobacterium* and *Lactobacillus* are the two most typical probiotics, and *Faecalibaculum, Blautia and Odoribacter* are also beneficial to hosts (shown in blue letter in Fig. 4B and Fig. S3A). Heat map of relative abundance of the top 50 bacteria at the genus taxonomic level showed that LAE significantly (*P* = 0.01) increased the relative abundance of *Bifidobacterium* on the 28th day compared with the water group, while AMP decreased it significantly (*P* = 0.046) on the 28th day. LAE elevated the relative abundance of *Faecalibaculum* (*P* = 0.0014), and AMP decreased it significantly (*P* = 0.0056) on the 28th day. Neither LAE nor AMP influenced the relative abundance of *Lactobacillus*, *Blautia* and *Odoribacter*. As for disease‐related gut microorganism (shown in red letter in Fig. 4b and Fig. S3B), on the 28th day, both AMP and LAE decreased the relative abundance of *Streptococcus* (*P* < 0.05), but only LAE inhibited *Mycoplasma* (*P* = 0.019). AMP significantly promoted the growth of *Prevotellaceae* (*P* = 0.0461 on the 28th day), *Parabacteroides* (*P* = 0.0275 on the 28th day) and *Enterorhabdus* (*P* = 0.0261 on the 14th day and *P* = 0.0253 on the 28th day). Moreover, the number of genera in the AMP group was significantly lower than that in the water group (Fig. 4C, *P* = 0.026) on the 14th day, indicated that AMP reduced the diversity of intestinal flora. There was no significant difference calculated among the three groups in the average number of classes, orders, families and species (Fig. S4). Hence, LAE was more beneficial to mouse gut microflora than ampicillin.

### LAE binds to acidic phospholipid phosphatidylserine and dissipates the bacterial membrane potential, causing membrane perturbations

**Figure 5:**
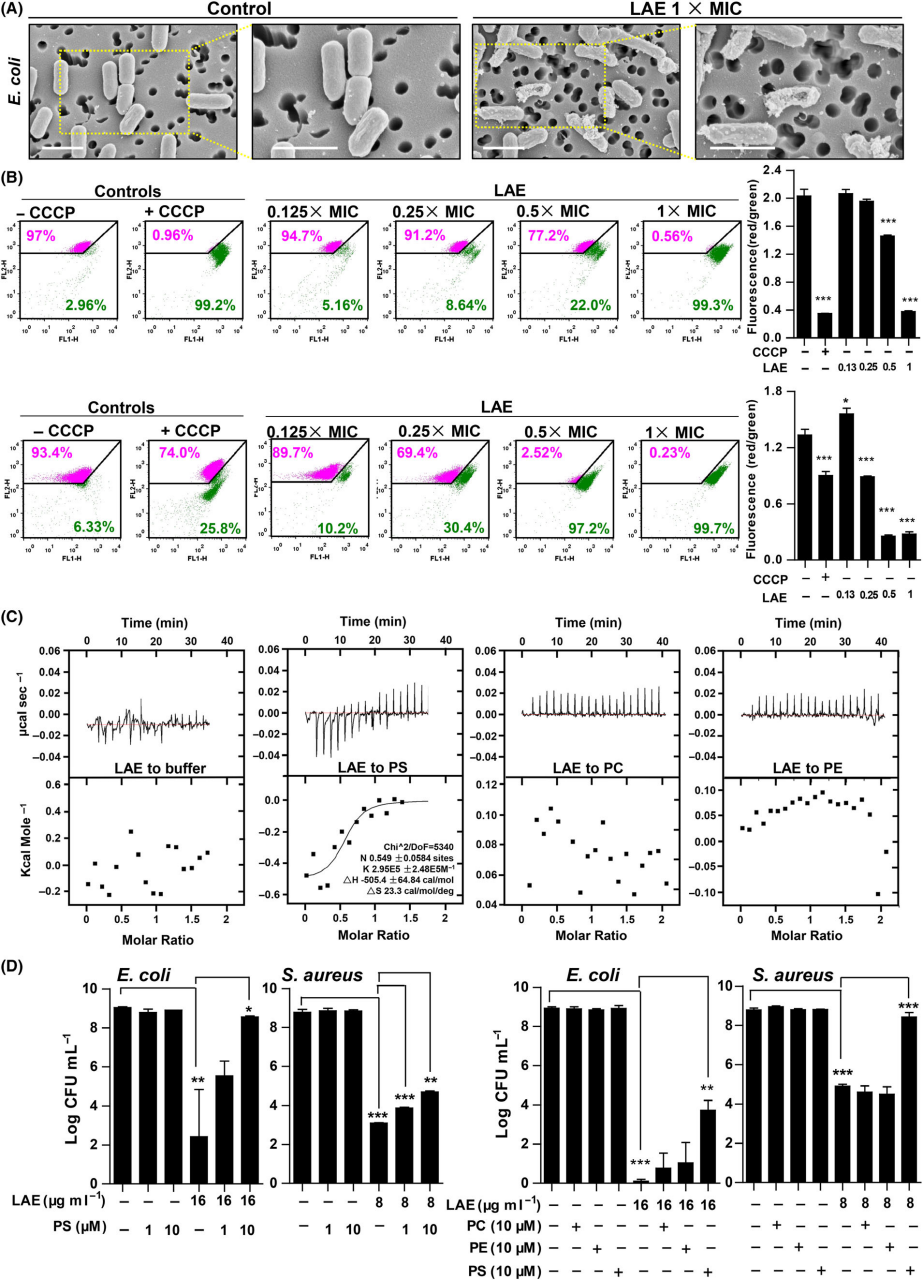
Antibacterial mechanism of LAE. LAE dissipates the bacterial membrane potential by binding PS, causing membrane perturbations and bacterial death. A. Scanning electron microscopy of *E. coli* treated with LAE or untreated controls. Scale bar, 1 μm. B. Membrane potential flow cytometric dot plots obtained after incubating *S. aureus* (B top) or *E. coli* (B bottom) with 30 µM DiOC_2_(3) for 1 h in the presence or absence of CCCP (a proton ionophore), and LAE at 0.125 ×,0.25 ×, 0.5 × and 1 × MIC. The controls where CCCP was either absent (− CCCP) or present (+ CCCP) represent the normal membrane potential state and fully depolarized state for bacteria respectively (*n* = 3). C. ITC measurements of the binding affinity of LAE to three kinds of phospholipids. D. Bacterial fluid was mixed with sterile ddH_2_O or different concentrations (10, 100 μM) of PS in the 96‐well plates. Then, 16 μg ml^−1^ of LAE (for *E. coli*) or 8 μg ml^−1^ of LAE (for *S. aureus*) was added. The plate was cultured in 37°C for 24 h. The number of viable bacteria of each well was estimated by diluting, plating and counting the colony‐forming units (CFU) on MHA plates. To test the difference in protective effect among PS, PC and PE, bacteria were mixed with the three kinds of phospholipids, respectively, at the same concentration, before treatment with LAE in a 96‐well plate. Viable bacterial number of each well was estimated after the plate was cultured in 37°C for 24 h (*n* = 3).

To observe the morphological changes resulting from LAE treatment, scanning electron microscope (SEM) analysis was performed on *E. coli* exposed to LAE at 1 × MIC or phosphate buffer. After 5 min of exposure to MIC concentration LAE, the bacteria exhibited external modifications including an irregular shape, significant rough surfaces, pore formation and cell debris (Fig. 5A). All these alterations on the outer bacterial envelope indicated that the cell membrane was one of the targets of LAE.

It was observed that the treatment of bacterial cells with LAE induced a shift towards a more depolarized population as concentration increased (Fig. 5B). These results suggested that the interaction of LAE with the bacteria resulted in membrane potential dissipation. The cell membrane defines the structure of both eukaryotic and prokaryotic cells, but LAE had lower cytotoxicity (Fig. 1E) and haemolysis of eukaryotic cells (Fig. 1F). Furthermore, the concentration of LAE needed for depolarization of the bacterial cell membrane was also different. 6.25 μg ml^−1^ of LAE depolarized bacterial cell membranes (Fig. S5A, B), while 50 μg ml^−1^ of LAE only caused slight depolarization of erythrocyte membranes (Fig. S5C). This phenomenon indicated that a difference between eukaryotic cell and prokaryotic cell membrane components was an antibacterial target of LAE.

Zwitterionic phospholipids, such as phosphatidylcholine (PC) or phosphatidylethanolamine (PE), are the main phospholipids in both eukaryotic and prokaryotic cell membranes, while acidic phospholipids with negative charges such as phosphatidylserine (PS) were distributed more on prokaryotic cell membrane surfaces (Matsuzaki, 1999; Zasloff, 2002; Lam *et al.*, 2016). Hence, the interaction between phospholipids and LAE was studied by isothermal titration calorimetry (ITC). When the acidic phospholipid PS was mixed with LAE, heat was released, and the KD value was 2.95E5 M. On the other hand, no interaction happened when the zwitterionic phospholipid PC or PE was mixed with LAE (Fig. 5C). The results suggested that LAE preferred to interact with acidic phospholipids.

Bacteria were treated with PS, PE or PC, respectively, prior to treating with LAE. The number of viable bacteria was not influenced by any of the phospholipids under control conditions, but was reduced by LAE, and this reduction was only rescued by the presence of PS. This phenomenon was observed in both *E. coli* and *S. aureus* (Fig, 5D), and suggested that the interaction between LAE and acidic phospholipids was involved in the process of the antibacterial effect of LAE.

## Discussion

Ethyl‐*N*‐dodecanoyl‐l‐arginate hydrochloride has been used as a food and cosmetic additive for more than 10 years; however, no published data are available on its induction of resistant bacteria, or on its antibacterial effects *in vivo*. In addition, the effect of LAE on animal growth performance and on the gut microbiome, as well as its antibacterial mechanism, also has not been clarified. Here, we find that LAE exhibits antibacterial activity in several infected animal models, and promotes body weight gain in mice, along with an increase in the relative abundance of *Bifidobacterium* in mouse guts. In addition, we found that LAE prefers to interact with acidic phospholipids of the cell membrane, suggesting that it has selective inhibitory effects on bacteria.

Ethyl‐*N*‐dodecanoyl‐l‐arginate hydrochloride is metabolized to l‐arginine, ornithine, CO_2_ and water in animals (Hawkins, *et al.*, 2009). l‐arginine is an abundant amino acid in tissue proteins and is used as feed additive to regulate nutrient metabolism and immune function in animals; therefore, it enhances the efficiency of feed utilization for protein accretion (He, *et al.*, 2009). Many other studies (Everett, *et al.*, 2017; Liu, *et al.*, 2017; Zhang, *et al.*, 2017) suggest that l‐arginine plays important roles in anti‐infection immunity including reducing bacterial invasion, regulating innate immune responses and protecting mice from infection. Here, our results indicate that LAE effectively combats bacterial infection in mice, ducklings and piglets. Combined with the results from other studies, LAE exerts antibacterial function *in vivo* by not only due to the direct inhibition of bacteria by LAE but also probably due to anti‐infection effects of the metabolite.

The thickness of the intestine wall (Dibner and Richards, 2005), animal vitality (Merks, *et al.*, 2012) and fat ratio affect the productivity of breeding animals. Evidence shows that β‐lactams make the chick intestine wall thinner and increase the permeability of nutrients to pass the wall, resulting in growth promotion (Coates, *et al.*, 1955). We did find that ampicillin significantly thinned the intestine wall; however, there was no growth promotion by ampicillin within 10 weeks in mice, and this phenomenon was also observed by other researchers in shorter time periods (Cho, *et al.*, 2012). LAE improved the weight gain of mice without decreasing the intestine wall thickness, influencing the body component ratio or animal vitality, and this phenomenon might be due to the amino acid it is metabolized to in mice, indicating that LAE is beneficial to animal growth in several ways.

Liver injury caused by drug treatments weakens digestive system functions, influencing animal productivity. It has been reported that β‐lactam antibiotics induce human liver injury in clinical practice (deLemos, *et al.*, 2016; Medina‐Caliz, *et al.*, 2016; Paech, *et al.*, 2017); however, few reports indicate the impact of ampicillin on animal livers. We found that ampicillin induced mouse hepatotoxicity, which is similar to hepatic granuloma in H&E staining. In addition, intestinal wall thinning is another injury ampicillin induced in mice. Taken together, our findings suggest LAE is a growth promoter with lower toxicity to animals than ampicillin.

Another side‐effect of antibacterial agents is that they unbalance the gut microbiota (Jernberg, *et al.*, 2010; Macfarlane, 2014). For instance, ampicillin and gentamicin cause a significant and immediate decrease in bacterial species richness and diversity (Johnson, *et al.*, 2015; Le Bastard, *et al.*, 2018). It has been shown that even short‐term antibiotic administration has long‐term impacts on the gut microbiome (Jakobsson, *et al.*, 2010). *Bifidobacterium*, the well‐recognized model probiotic bacterial genera in gut, is beneficial to host with effects on absorption and utilization of feed, increasing the productivity of various animals. *Faecalibacterium* is the most abundant bacterium in the human intestinal microbiota of healthy adults which is thought to confer anti‐inflammatory benefits (Miquel, *et al.*, 2013). Intestinal *Blautia* reduced death from graft‐versus‐host disease (Jenq, *et al.*, 2015). *Odoribacter* shows great potential to maintain the intestine or immune system by producing sulfonolipids (Walker, *et al.*, 2017). In contrast to these beneficial microbes, *Mycoplasmas* cause respiratory and reproductive system infection in hosts (Gautier‐Bouchardon, 2018; Zhou, *et al.*, 2018). *Streptococcus* is a host‐adapted bacterial pathogen that is among the leading infectious causes of host morbidity and mortality (Barnett, *et al.*, 2015). *Enterorhabdus* is related to inflammatory diseases (Clavel, *et al.*, 2009; Yang, *et al.*, 2015). *Parabacteroides* enhances colitis in mice (Dziarski, *et al.*, 2016). *Prevotellaceae* promotes inflammation‐associated intestinal carcinogenesis (Gagliani, *et al.*, 2014). In the present study, LAE elevated the probiotic microorganism levels, while ampicillin promoted the growth of several disease‐related microorganisms. Ampicillin reduces the richness of the gut microbiome at the genus level as well. These results suggest that LAE enhances animal health partly via increasing the proportion of health‐promoting bacteria and is more beneficial to gut health than ampicillin.

Membrane integrity targeting compounds, for example polymyxins and LAE (Rodriguez, *et al.*, 2004), destroy the first line of bacterial defence which prevents bacteria from developing drug resistance. Consistent with these findings, there was no resistance in *E. coli* induced by LAE, while the bacteria became insensitive to several kinds of antibiotics which target biomacromolecules. This phenomenon might be due to the membrane targeting antibacterial mechanism of LAE.

The membrane is a cellular structure common to both eukaryotic and prokaryotic cells. However, LAE shows excellent antibacterial activity with low toxicity against mammalian cells, and the concentration of LAE required for depolarization of erythrocyte and bacterial cell membranes is different. To find the cause of these differences, we focused on the acidic phospholipid phosphatidylserine, which is a negatively charged molecule with a different local distribution between eukaryotic and prokaryotic cell membranes (Matsuzaki, 1999; Zasloff, 2002; Lam *et al.*, 2016). Hence, we supposed that phosphatidylserine interacts with positively charged LAE and participates in the antibacterial process of LAE. The ITC results show that LAE binds to phosphatidylserine instead of neutral phospholipids such as phosphatidylcholine or phosphatidylethanolamine, which are the main membrane phospholipids in both eukaryotic and prokaryotic cells. Furthermore, only phosphatidylserine protected bacteria from LAE‐induced death and the protection was in a dose‐dependent manner. Taken together, we suggest that negatively charged acid phospholipids attract positively charged LAE to the bacterial membrane surface resulting in membrane depolarization and further leading to membrane disintegration and bacterial death. However, there are thousands of proteins embedded in or on the surface of the phospholipid bilayer, so the effect of LAE on membrane proteins remains undetermined in our work and needs further investigation.

In conclusion, LAE binds to acid phospholipids and depolarizes the bacterial membrane resulting in bacterial death with low drug resistance. LAE exhibits antibacterial activity *in vivo* and promotes growth with potentially beneficial modifications to the gut microbiota. As a result, LAE is not only a potential veterinary drug for bacterial infection but also a potential low‐risk feed additive for animal growth.

## Experimental procedures

### Cells and bacteria

Mouse myoblasts (C2C12) and human fibroblast (HaF) were purchased from ATCC (Manassas, VA, USA). Immortalized human keratinocytes (HaCaTs) were purchased from the Type Culture Collection of the Chinese Academy of Sciences, Shanghai, China. Cells were maintained in DMEM supplemented with 10% FBS and 1% penicillin/streptomycin. *E. coli* ATCC 25922 and *S. aureus* ATCC 29213 were purchased from ATCC (Manassas, VA, USA). *R. anatipestifer* strain was isolated from an infected duck farm in China by the Shanghai Veterinary Research Institute, Chinese Academy of Agricultural Sciences. All bacteria were cultured in Mueller‐Hinton broth (MHB; Oxoid, Basingstoke, Hants, UK).

### Mouse husbandry

Male BALB/c mice were purchased from the Shanghai Laboratory Animal Company (Shanghai, China). Mice used in these experiments were housed under pathogen‐free conditions and were maintained in accordance with institutional guidelines. All experimental protocols were approved by the Animal Investigation Committee of East China Normal University (No. m20190212).

### Measurement of Minimum Inhibitory Concentration (MIC) and Minimum Bactericidal Concentration (MBC)

The MIC was determined by the broth microdilution procedure according to the Clinical & Laboratory Standards Institute (CLSI) guidelines. The broth microdilution method involved a twofold serial dilution of range of 3.12–100 μg ml^−1^. Strains were grown in MHB at 37°C in an ambient atmosphere. The MIC was determined as the lowest concentration of compound that inhibits visible growth following a 24 h of incubation period. For MBC assays, the bacterial suspensions were diluted in PBS solution and plated on MHB agar plates. The plates were incubated for 24 h at 37°C, and the CFU was counted. MBC was defined as the lowest concentration of drug that killed ≥ 99.9% initial inoculums after 24 h of incubation at 37°C. Results presented are representative of at least two experiments.

### Time‐kill assays

Bacteria cells at exponential phase were diluted with Tryptone Soya Broth (TSB). Approximately 5 × 10^5^ CFU ml^−1^ organisms were inoculated into each member of a series of test tubes containing twofold concentration increments of drug. Cells were incubated at 37°C with shaking at 220 rpm. At 0, 0.5, 1, 1.5, 2 h of time points, viable bacterial numbers were estimated by diluting, plating and counting the colony‐forming unit (CFU) on Tryptone Soya Broth agar (TSA) plates.

### Resistance studies

Resistance studies were performed by using a modified method described by Ling et. al (Ling, *et al.*, 2015). For resistance development by sequential passaging, *S. aureus* ATCC 29213 or *E. coli* ATCC 25922 cells at exponential phase were diluted with MHB. At least 10^7^ organisms were inoculated into each member of a series of test tubes containing twofold concentration increments of drug. Cells were incubated at 37°C with shaking and passaged at 24 h of intervals in the presence of LAE or chloramphenicol at different concentrations. Bacteria were sequentially cultured in the presence of different concentrations of LAE in order to increase the probability of obtaining resistant mutants. *S. aureus* (ATCC 29213) or *E. coli* ATCC 25922 cells were grown in 2 ml of MHB containing LAE at different concentrations. Cells were added to LAE present at 0.25 × MIC, 0.5 × MIC, 1 × MIC, 2 × MIC and 4 × MIC. At 24 h of intervals, the cultures were checked for growth. Cultures from the second highest concentrations that allowed growth were diluted 1:100 into fresh media containing 0.25 × MIC, 0.5 × MIC, 1 × MIC, 2 × MIC and 4 × MIC of LAE. This serial passaging was repeated daily for 30 days.

### Cytotoxicity

The cells were seeded into 96‐well plates at a density of 5000 cells per well and washed with sterile PBS once to remove the unattached cells after 24 h. 100 μl of serially diluted LAE was added into the adherent cells and incubated for 48 h. Cytotoxicity was determined using a cell viability assay (CCK8, Cell Counting Kit‐8, Kumamoto, Japan). 10 μl CCK8 was added into the cells and mixed gently, and the plate was cultivated at 37°C and read at 450 nm until the OD_450_ of controls was between 0.8 and 1.2. The concentration of the test compound that reduced the cell viability by 50% (IC_50_) was determined by non‐linear regression analysis using GraphPad Prism 5.0 software. Two independent experiments were carried out, and three samples per test were taken for statistical analysis.

### Haemolytic activity

Two percentage of red blood cell suspension was added to the wells of a 96‐well U‐bottomed plate. LAE was serially diluted twofold in PBS and added to the wells resulting in a final concentration ranging from 4 to 256 μg ml^−1^. 0.5% Triton X‐100 (Sangon, Shanghai, China) was used as a positive control, while 0.01M PBS was used as a negative control. After 1 h, the plate was centrifuged and the absorbance value of the supernatant at 450 nm was measured. Two independent experiments were carried out, and three samples per test were taken for statistical analysis.

### Peritonitis mouse model

Six‐ to 8‐week‐old BALB/c female mice were chosen for induction of peritonitis to mimic bacterial infection. To count the number of bacteria in peritoneal fluid, mice were challenged with 10^8^ CFU bacteria through intraperitoneal (i.p.) injection. An hour later, mice were divided randomly into different groups and treated with indicated concentrations of antibiotics or LAE. 24 h after infection, the peritoneal cavity of each mouse was washed with 2 ml PBS, and peritoneal fluid was collected. The number of survival pathogens was calculated on TSB plates. Another three groups were divided and challenged with 10^9^ CFU bacteria through intraperitoneal injection 1 h before treatment with drugs as described above, and the mice were checked every 2 h to obtain a survival curve.

### Enzyme‐linked immunosorbent assay (ELISA)

To test the inflammatory factor level from mouse peritoneal fluid as described above, secretion of IL‐1β and TNF‐α was measured by the use of mouse OptEIA enzyme‐linked immunosorbent assay (ELISA) kits (BD Biosciences, San Diego, CA, USA).

### Duckling R. anatipestifer infection model

The effects of LAE on *R. anatipestifer*‐infected ducklings were tested in the Shanghai Veterinary Research Institute (Chinese Academy of Agricultural Sciences). 60 7‐day‐old Cherry Valley ducklings were randomly divided into 6 groups (*n* = 10). Each drug‐treated group (except for the uninfected group) was intramuscularly injected with 0.2 ml of *R. anatipestifer* diluted to 5 × 10^6^ CFU 8 h after the first drug administration. Ducklings were treated orally with drug at the indicated doses 3 times per day for 3 days. The survival rate of ducklings was recorded for 7 days.

### Piglet yellow scour assay

A pig farm with outbreak of yellow scour in the Henan province of China (Henan Guangan Group) was selected as the experimental site. 118 ill piglets (3–13 days of age) were treated with 10 mg kg^‐1^, 50 mg kg^‐1^ LAE oral administration (p.o.) or 50 mg kg^‐1^ gentamicin intramuscular injection, (i.m.) three times per day for four days. The number of surviving piglets of each group was recorded.

### Mouse gut microbiome analysis

Male BALB/c mice were obtained on day 21 of life from Shanghai Laboratory Animal Company (Shanghai, China). Mice were weighed at the beginning and distributed ten per group to achieve equal mean weights in each group and were housed five per cage. Beginning on day 28 of life, mice were given untreated water, or water containing ampicillin or water containing LAE. Doses were adapted from the FDA Green Book and generally were in the mid‐range of ampicillin approved for the use in agriculture. To simplify administration, the drugs were added to drinking water at a dose of 1 μg per g body weight of the mice, based on the calculation that daily water intake is proportional to body weight. Two times a week, each mouse was weighed. The growth rate of group LAE relative to group water is calculated as follows:

$$\%\;\text{weight}\;\text{gain}\;=\;\frac{\text{average}\;\text{weight}\;\text{gain}\;\text{of}\;\text{LAE}\;\text{group}\;-\;\text{average}\;\text{weight}\;\text{gain}\;\text{of}\;\text{water}\;\text{group}}{\text{average}\;\;\text{weight}\;\text{gain}\;\text{of}\;\text{water}\;\text{group}}\times 100.$$

Faecal pellets were collected every week and stored at −20°C until DNA extraction. The v3–v4 region of the 16S rDNA gene was amplified using forward primer (5’‐ACTCCTACGGGAGGCAGCA‐3’) and reverse primer (5’‐GGACTACHVGGGTWTCTAAT‐3’). The Illumina MiSeq sequencing library was prepared using amplified products as template. The reference database for sequencing analysis was Silva. Body composition was determined using a body fat measurement instrument (MEG‐Accufat) every week. All mice were sacrificed and hearts, livers, spleen, lungs and kidneys were isolated and weighed. For pathological investigation, all tissues were immediately fixed in 4% paraformaldehyde solution.

### Indirect calorimetry

BALB/c mice were singly housed in metabolic cages under a 12 h of light/dark cycle. Mice had unrestricted access to feed and ampicillin, LAE or control water, as appropriate. There was a 1‐day acclimatization period, followed by 2 days of measurements. Data on VO_2_ and VCO_2_ were recorded every 10 or 11 min for a 48 h of period at 23°C. Physical activity was measured by infrared light detector.

### Isothermal titration calorimetry (ITC)

ITC measurements were performed with a MicroCal 200 Instrument (Huang, *et al.*, 2016). A total of 80 μl of LAE (1 mM) was injected (2 μl for each injection) into the reaction cell containing 400 μl of phospholipids (0.1 mM) at 25°C. The titrant was injected at 5 min of intervals to ensure that the titration peak returned to the baseline before the next injection.

### Membrane potential assay

Membrane potential was determined by flow cytometry using a BacLight Bacterial^TM^ Membrane Potential Kit (Invitrogen B34950) (Lam *et al.*, 2016). *E. coli* cells were cultured to mid‐log phase. Viable cells were then diluted to 2.5 × 10^7^ cells ml^−1^ in PBS and treated with different concentrations (0.125 ×, 0. 25 ×, 0. 5 ×, 1 × MIC) of LAE. CCCP at a final concentration of 5 μM was performed as positive control. Before a 1 h of incubation at room temperature, 30 μM DiOC_2_(3) was added to all samples. Membrane potential was determined by a cell flow cytometer (BD FACSCalibur ^TM^) as the ratio of cells that exhibited red fluorescence to those that displayed green fluorescence. Data were representative of two independent assays completed in duplicate.

### Phosphatidylserine protection assay

Bacterial cells in mid‐exponential phase were diluted to 1.25 × 10^8^ cells ml^−1^ in MHB. 80 μl of bacterial fluid was mixed with 20 μl sterile ddH_2_O or different concentrations (10, 100 μM) of PS in the 96‐well plates. Then, 100 μl of MHB containing 16 μg ml^−1^ of LAE (for *E. coli*) or 8 μg ml^−1^ of LAE (for *S. aureus*) was added to the wells. The plate was cultured in 37°C for 24 h. The number of viable bacteria of each well was estimated by diluting, plating and counting the colony‐forming units (CFU) on MHA plates. To test the difference in protective effect among PS, PC and PE, bacteria were mixed with the three kinds of phospholipids, respectively, at the same concentration, before treatment with LAE in a 96‐well plate. Viable bacterial number of each well was estimated after the plate was cultured in 37°C for 24 h.

### Statistics

Grouped data are expressed as mean ± SD. Significance between groups was analysed by one‐way ANOVA or Student's *t*‐test using graphpad prism 5.0 (GraphPad Soft, San Diego, CA, USA). Differences were considered significant when *P* < 0.05 (*), *P* < 0.01 (**) or *P* < 0.001 (***).

## Conflict of interest

None declared.

## Supporting information


**Fig. S1.** There was no different in body fat ratio (A), body lean ratio (B), horizontal activity (C), standing counts (D), or organ index (E), respiratory exchange rate (F) and heat consumption rate (G) between the three groups.

**Fig. S2.** Both LAE and Ampicillin show non‐toxicity to mouse heart, spleen, lung and kidney. The influence of LAE on mouse organs. BALB/c mice were exposed to LAE or Ampicillin (AMP) in drinking water, or kept as untreated controls (Water) for 10 Weeks (*n* = 10). The mouse organs were harvested, photographed (A), sectioned, and stained, and stained with H&E (B). Scale bar, 100 μm.

**Fig. S3.** The influence of LAE and ampicillin on the relative abundance of several probiotic or disease‐related gut microorganism genera and families. The relative abundance of (A) probiotic microorganisms (shown in blue letters) *Bifidobacterium*, *faecalibaculum*, *Lactobacillus, Blautia, Odoribactor, *and (B) disease‐related microorganisms (shown in red letters) *Prevotellaceae*, *Streptococcus, Mycoplama, paeabacteroids* and *Enterorhabsus *in water, ampicillin (1 mg kg^−1^), and LAE (1 mg kg^−1^) treated groups on day 0/14/28 of drug administration (*n* = 10).

**Fig. S4.** The influence of LAE on the number of mice gut microbial groups at each taxonomic level. The number of microbial groups (*n* = 10) at the class (A), order (B), family (C) and species (D) taxonomic level.

**Fig. S5.** Depolarization of eukaryotic and prokaryotic cell membranes induced by LAE. *E. coli *(A) or *S. aureus *(B) or rat blood cells (C) were incubated with 0.4 μM DiOC_3_(5) for 1 h and 100 m M KCL was added into the bacterial suspension to equilibrate the intracellular and external K^+ ^concentrations. Then PBS, LAE or antibiotics in indicated concentrations was added to the samples, respectively. The fluorescence of each sample was monitored using a fluorescence spectrophotometer (Hitachi, Japan) at an excitation wavelength of 622 nm and an emission wavelength of 670 nm.

**Table S1.** The effect of LAE on piglets yellow scour.

## References

[mbt213514-bib-0001] World Health Organization (2018) Global Health Estimates 2016: Deaths by Cause, Age, Sex, by Country and by Region, 2000‐2016. Geneva, Switzerland: World Health Organization.

[mbt213514-bib-0002] Barnett, T.C. , Cole, J.N. , Rivera‐Hernandez, T. , Henningham, A. , Paton, J.C. , Nizet, V. , and Walker, M.J. (2015) Streptococcal toxins: role in pathogenesis and disease. Cell Microbiol 17: 1721–1741. 26433203 10.1111/cmi.12531

[mbt213514-bib-0003] Butaye, P. , Devriese, L.A. , and Haesebrouck, F. (2003) Antimicrobial growth promoters used in animal feed: effects of less well known antibiotics on gram‐positive bacteria. Clin Microbiol Rev 16: 175–188. 12692092 10.1128/CMR.16.2.175-188.2003 PMC153145

[mbt213514-bib-0004] Casey, P.G. , Gardiner, G.E. , Casey, G. , Bradshaw, B. , Lawlor, P.G. , Lynch, P.B. , *et al* (2007) A five‐strain probiotic combination reduces pathogen shedding and alleviates disease signs in pigs challenged with *Salmonella enterica* Serovar Typhimurium. Appl Environ Microbiol 73: 1858–1863. 17261517 10.1128/AEM.01840-06 PMC1828830

[mbt213514-bib-0005] Chee‐Sanford, J.C. , Mackie, R.I. , Koike, S. , Krapac, I.G. , Lin, Y.F. , Yannarell, A.C. , *et al* (2009) Fate and transport of antibiotic residues and antibiotic resistance genes following land application of manure waste. J Environ Qual 38: 1086–1108. 19398507 10.2134/jeq2008.0128

[mbt213514-bib-0006] Cho, I. , Yamanishi, S. , Cox, L. , Methe, B.A. , Zavadil, J. , Li, K. , *et al* (2012) Antibiotics in early life alter the murine colonic microbiome and adiposity. Nature 488: 621–626. 22914093 10.1038/nature11400 PMC3553221

[mbt213514-bib-0007] Clavel, T. , Charrier, C. , Braune, A. , Wenning, M. , Blaut, M. , and Haller, D. (2009) Isolation of bacteria from the ileal mucosa of TNFdeltaARE mice and description of *Enterorhabdus mucosicola* gen. nov., sp. nov. Int J Syst Evol Microbiol 59: 1805–1812. 19542111 10.1099/ijs.0.003087-0

[mbt213514-bib-0008] Coates, M.E. , Davies, M.K. , and Kon, S.K. (1955) The effect of antibiotics on the intestine of the chick. Br J Nutri 9: 110–119. 10.1079/bjn19550016 14351666

[mbt213514-bib-0009] Danzeisen, J.L. , Clayton, J.B. , Huang, H. , Knights, D. , McComb, B. , Hayer, S.S. , and Johnson, T.J. (2015) Temporal relationships exist between cecum, ileum, and litter bacterial microbiomes in a commercial turkey flock, and subtherapeutic penicillin treatment impacts ileum bacterial community establishment. Front Veterinary Sci 2: 56. 10.3389/fvets.2015.00056 PMC4672264 26664983

[mbt213514-bib-0010] Dibner, J.J. , and Richards, J.D. (2005) Antibiotic growth promoters in agriculture: history and mode of action. Poult Sci 84: 634–643. 15844822 10.1093/ps/84.4.634

[mbt213514-bib-0011] Dziarski, R. , Park, S.Y. , Kashyap, D.R. , Dowd, S.E. , and Gupta, D. (2016) Pglyrp‐regulated gut microflora prevotella falsenii, parabacteroides distasonis and bacteroides eggerthii enhance and *Alistipes finegoldii* attenuates colitis in mice. PLoS ONE 11: e0146162. 26727498 10.1371/journal.pone.0146162 PMC4699708

[mbt213514-bib-0012] Everett, J. , Turner, K. , Cai, Q. , Gordon, V. , Whiteley, M. , and Rumbaugh, K. (2017) Arginine is a critical substrate for the pathogenesis of *Pseudomonas aeruginosa* in burn wound infections. MBio 8: e02160-16. 28292986 10.1128/mBio.02160-16 PMC5350470

[mbt213514-bib-0013] Gadde, U. , Kim, W.H. , Oh, S. T. , and Lillehoj, H. S. (2017) Alternatives to antibiotics for maximizing growth performance and feed efficiency in poultry: a review. Anim Health Res Rev 18: 26–45. 28485263 10.1017/S1466252316000207

[mbt213514-bib-0014] Gagliani, N. , Hu, B. , Huber, S. , Elinav, E. , and Flavell, R.A. (2014) The fire within: microbes inflame tumors. Cell 157: 776–783. 24813605 10.1016/j.cell.2014.03.006

[mbt213514-bib-0015] Gao, P. , Ma, C. , Sun, Z. , Wang, L. , Huang, S. , Su, X. , *et al* (2017) Feed‐additive probiotics accelerate yet antibiotics delay intestinal microbiota maturation in broiler chicken. Microbiome 5: 91. 28768551 10.1186/s40168-017-0315-1 PMC5541433

[mbt213514-bib-0016] Gautier‐Bouchardon, A. V. (2018) Antimicrobial resistance in *Mycoplasma* spp. Microbiology Spectrum 6: ARBA-0030-2018. 10.1128/microbiolspec.arba-0030-2018 PMC11633602 30003864

[mbt213514-bib-0017] Gullberg, E. , Cao, S. , Berg, O.G. , Ilback, C. , Sandegren, L. , Hughes, D. , and Andersson, D.I. (2011) Selection of resistant bacteria at very low antibiotic concentrations. Plos Pathog 7: e1002158. 21811410 10.1371/journal.ppat.1002158 PMC3141051

[mbt213514-bib-0018] de Gunzburg, J. , Ghozlane, A. , Ducher, A. , Le Chatelier, E. , Duval, X. , Ruppe, E. , *et al* (2018) Protection of the human gut microbiome from antibiotics. J Infect Dis 217: 628–636. 29186529 10.1093/infdis/jix604 PMC5853327

[mbt213514-bib-0019] de Lemos, A.S. , Ghabril, M. , Rockey, D.C. , Gu, J. , Barnhart, H.X. , Fontana, R.J. , *et al* (2016) Amoxicillin‐clavulanate‐induced liver injury. Dig Dis Sci 61: 2406–2416. 27003146 10.1007/s10620-016-4121-6 PMC4945382

[mbt213514-bib-0020] Hawkins, D.R. , Rocabayera, X. , Ruckman, S. , Segret, R. , and Shaw, D. (2009) Metabolism and pharmacokinetics of ethyl N(alpha)‐lauroyl‐L‐arginate hydrochloride in human volunteers. Food Chem Toxicol 47: 2711–2715. 19651183 10.1016/j.fct.2009.07.028

[mbt213514-bib-0021] He, Q. , Kong, X. , Wu, G. , Ren, P. , Tang, H. , Hao, F. , *et al* (2009) Metabolomic analysis of the response of growing pigs to dietary L‐arginine supplementation. Amino Acids 37: 199–208. 18989615 10.1007/s00726-008-0192-9

[mbt213514-bib-0022] Hendrickx, A.P. , Top, J. , Bayjanov, J.R. , Kemperman, H. , Rogers, M.R. , Paganelli, F.L. , *et al* (2015) Antibiotic‐driven dysbiosis mediates intraluminal agglutination and alternative segregation of enterococcus faecium from the intestinal epithelium. MBio 6: e01346‐01315. 26556272 10.1128/mBio.01346-15 PMC4659461

[mbt213514-bib-0023] Huang, J. , Shang, K. , Kashif, J. , and Wang, L. (2015) Genetic diversity of *Streptococcus suis* isolated from three pig farms of China obtained by acquiring antibiotic resistance genes. J Sci Food Agric 95: 1454–1460. 25060787 10.1002/jsfa.6841

[mbt213514-bib-0024] Huang, P.J. , Wang, F. , and Liu, J. (2016) Liposome/graphene oxide interaction studied by isothermal titration calorimetry. Langmuir 32: 2458–2463. 26908113 10.1021/acs.langmuir.6b00006

[mbt213514-bib-0025] Jakobsson, H.E. , Jernberg, C. , Andersson, A.F. , Sjolund‐Karlsson, M. , Jansson, J. K. , and Engstrand, L. (2010) Short‐term antibiotic treatment has differing long‐term impacts on the human throat and gut microbiome. PLoS ONE 5: e9836. 20352091 10.1371/journal.pone.0009836 PMC2844414

[mbt213514-bib-0026] Jenq, R.R. , Taur, Y. , Devlin, S.M. , Ponce, D.M. , Goldberg, J.D. , Ahr, K.F. , *et al* (2015) Intestinal blautia is associated with reduced death from graft‐versus‐host disease. Biol Blood Marrow Transplant 21: 1373–1383. 25977230 10.1016/j.bbmt.2015.04.016 PMC4516127

[mbt213514-bib-0027] Jernberg, C. , Lofmark, S. , Edlund, C. , and Jansson, J.K. (2010) Long‐term impacts of antibiotic exposure on the human intestinal microbiota. Microbiology 156: 3216–3223. 20705661 10.1099/mic.0.040618-0

[mbt213514-bib-0028] Johnson, L.P. , Walton, G.E. , Psichas, A. , Frost, G.S. , Gibson, G.R. , and Barraclough, T. G. (2015) Prebiotics modulate the effects of antibiotics on gut microbial diversity and functioning in vitro. Nutrients 7: 4480–4497. 26053617 10.3390/nu7064480 PMC4488797

[mbt213514-bib-0029] Kofteridis, D.P. , Malliotakis, P. , Maraki, S. , Christofaki, M. , and Samonis, G. (2009) Impact of prolonged treatment with linezolid on the human gut flora. Int J Infect Dis 13: e313–315. 19395298 10.1016/j.ijid.2009.02.001

[mbt213514-bib-0030] Kupferschmidt, K. (2016) Resistance fighters. Science 352: 758–761. 27174968 10.1126/science.352.6287.758

[mbt213514-bib-0031] Lam, S.J. , O'Brien‐Simpson, N.M. , Pantarat, N. , Sulistio, A. , Wong, E.H. , Chen, Y.Y. , *et al* (2016) Combating multidrug‐resistant Gram‐negative bacteria with structurally nanoengineered antimicrobial peptide polymers. Nat Microbiol 1: 16162. 27617798 10.1038/nmicrobiol.2016.162

[mbt213514-bib-0033] Le Bastard, Q. , Ward, T. , Sidiropoulos, D. , Hillmann, B.M. , Chun, C.L. , Sadowsky, M.J. , *et al* (2018) Fecal microbiota transplantation reverses antibiotic and chemotherapy‐induced gut dysbiosis in mice. Sci Rep‐Uk 8: 6219. 10.1038/s41598-018-24342-x PMC5906603 29670191

[mbt213514-bib-0034] Levy, S.B. , and Marshall, B. (2004) Antibacterial resistance worldwide: causes, challenges and responses. Nat Med 10: S122–S129. 15577930 10.1038/nm1145

[mbt213514-bib-0035] Ling, L.L. , Schneider, T. , Peoples, A.J. , Spoering, A.L. , Engels, I. , Conlon, B.P. , *et al* (2015) A new antibiotic kills pathogens without detectable resistance. Nature 517: 455–459. 25561178 10.1038/nature14098 PMC7414797

[mbt213514-bib-0036] Liu, G. , Ren, W. , Fang, J. , Hu, C.A. , Guan, G. , Al‐Dhabi, N.A. , *et al* (2017) L‐Glutamine and L‐arginine protect against enterotoxigenic *Escherichia coli* infection via intestinal innate immunity in mice. Amino Acids 49: 1945–1954. 28299479 10.1007/s00726-017-2410-9

[mbt213514-bib-0037] Macfarlane, S. (2014) Antibiotic treatments and microbes in the gut. Environ Microbiol 16: 919–924. 24471523 10.1111/1462-2920.12399

[mbt213514-bib-0038] Matsuzaki, K. (1999) Why and how are peptide‐lipid interactions utilized for self‐defense? Magainins and tachyplesins as archetypes, Biochimica et biophysica acta 1462: 1–10. 10590299 10.1016/s0005-2736(99)00197-2

[mbt213514-bib-0039] Medina‐Caliz, I. , Robles‐Diaz, M. , Garcia‐Munoz, B. , Stephens, C. , Ortega‐Alonso, A. , Garcia‐Cortes, M. , *et al* (2016) Definition and risk factors for chronicity following acute idiosyncratic drug‐induced liver injury. J Hepatol 65: 532–542. 27184533 10.1016/j.jhep.2016.05.003 PMC7458366

[mbt213514-bib-0040] Merks, J.W. , Mathur, P.K. , and Knol, E.F. (2012) New phenotypes for new breeding goals in pigs. Animal 6: 535–543. 22436267 10.1017/S1751731111002266

[mbt213514-bib-0041] Miquel, S. , Martín, R. , Rossi, O. , Bermúdez‐Humarán, L.G. , Chatel, J.M. , Sokol, H. , *et al* (2013) Faecalibacterium prausnitzii and human intestinal health. Curr Opin Microbiol 16: 255–261. 23831042 10.1016/j.mib.2013.06.003

[mbt213514-bib-0042] Muriel‐Galet, V. , Lopez‐Carballo, G. , Gavara, R. , and Hernandez‐Munoz, P. (2012) Antimicrobial food packaging film based on the release of LAE from EVOH. Int J Food Microbiol 157: 239–244. 22640726 10.1016/j.ijfoodmicro.2012.05.009

[mbt213514-bib-0043] Paech, F. , Messner, S. , Spickermann, J. , Wind, M. , Schmitt‐Hoffmann, A.H. , Witschi, A.T. , *et al* (2017) Mechanisms of hepatotoxicity associated with the monocyclic beta‐lactam antibiotic BAL30072. Arch Toxicol 91: 3647–3662. 28536862 10.1007/s00204-017-1994-x

[mbt213514-bib-0044] Pallav, K. , Dowd, S.E. , Villafuerte, J. , Yang, X. , Kabbani, T. , Hansen, J. , *et al* (2014) Effects of polysaccharopeptide from *Trametes versicolor* and amoxicillin on the gut microbiome of healthy volunteers: a randomized clinical trial. Gut Microbes 5: 458–467. 25006989 10.4161/gmic.29558

[mbt213514-bib-0045] Redondo, L.M. , Dominguez, J.E. , Rabinovitz, B.C. , Redondo, E.A. , and Fernandez Miyakawa, M.E. (2015) Hydrolyzable and condensed tannins resistance in Clostridium perfringens. Anaerobe 34: 139–145. 26037239 10.1016/j.anaerobe.2015.05.010

[mbt213514-bib-0046] Rodriguez, E. , Seguer, J. , Rocabayera, X. , and Manresa, A. (2004) Cellular effects of monohydrochloride of L‐arginine, N‐lauroyl ethylester (LAE) on exposure to *Salmonella typhimurium* and *Staphylococcus aureus* . J Appl Microbiol 96: 903–912. 15078505 10.1111/j.1365-2672.2004.02207.x

[mbt213514-bib-0047] Ruckman, S.A. , Rocabayera, X. , Borzelleca, J.F. , and Sandusky, C.B. (2004) Toxicological and metabolic investigations of the safety of N‐α‐Lauroyl‐l‐arginine ethyl ester monohydrochloride (LAE). Food Chem Toxicol 42: 245–259. 14667471 10.1016/j.fct.2003.08.022

[mbt213514-bib-0048] Samli, H.E. , Senkoylu, N. , Koc, F. , Kanter, M. , and Agma, A. (2007) Effects of *Enterococcus faecium* and dried whey on broiler performance, gut histomorphology and intestinal microbiota. Arch Anim Nutr 61: 42–49. 17361947 10.1080/17450390601106655

[mbt213514-bib-0049] Schwartz, S. , Clare, R. , Devereux, K. , and Sheung, C. F. (1997) Pharmacokinetics, disposition and metabolism of 546C88 (L‐NG‐methylarginine hydrochloride) in rat and dog. Xenobiotica 27: 1259–1271. 9460231 10.1080/004982597239840

[mbt213514-bib-0050] Shi, J. , Yu, X. , Zhang, M. , Lu, S. , Wu, W. , Wu, J. , and Xu, J. (2011) Potential risks of copper, zinc, and cadmium pollution due to pig manure application in a soil‐rice system under intensive farming: a case study of Nanhu, China. J Environ Qual 40: 1695–1704. 22031551 10.2134/jeq2010.0316

[mbt213514-bib-0051] Turroni, F. , Bottacini, F. , Foroni, E. , Mulder, I. , Kim, J.H. , Zomer, A. , *et al* (2010) Genome analysis of Bifidobacterium bifidum PRL2010 reveals metabolic pathways for host‐derived glycan foraging. Proc Natl Acad Sci USA 107: 19514–19519. 20974960 10.1073/pnas.1011100107 PMC2984195

[mbt213514-bib-0052] Turroni, F. , Ventura, M. , Butto, L.F. , Duranti, S. , O'Toole, P.W. , Motherway, M.O. , and van Sinderen, D. (2014) Molecular dialogue between the human gut microbiota and the host: a Lactobacillus and Bifidobacterium perspective. Cell Mol Life Sci 71: 183–203. 23516017 10.1007/s00018-013-1318-0 PMC11113728

[mbt213514-bib-0053] Walker, A. , Pfitzner, B. , Harir, M. , Schaubeck, M. , Calasan, J. , Heinzmann, S.S. , *et al* (2017) Sulfonolipids as novel metabolite markers of Alistipes and Odoribacter affected by high‐fat diets. Sci Rep 7: 11047. 28887494 10.1038/s41598-017-10369-z PMC5591296

[mbt213514-bib-0054] Wang, Y. , Tian, G.B. , Zhang, R. , Shen, Y. , Tyrrell, J.M. , Huang, X. , *et al* (2017) Prevalence, risk factors, outcomes, and molecular epidemiology of mcr‐1‐positive Enterobacteriaceae in patients and healthy adults from China: an epidemiological and clinical study. Lancet Infect Dis 17: 390–399. 28139431 10.1016/S1473-3099(16)30527-8

[mbt213514-bib-0055] Wenzel, M. , Chiriac, A.I. , Otto, A. , Zweytick, D. , May, C. , Schumacher, C. , *et al* (2014) Small cationic antimicrobial peptides delocalize peripheral membrane proteins. Proc Natl Acad Sci USA 111: E1409–1418. 24706874 10.1073/pnas.1319900111 PMC3986158

[mbt213514-bib-0056] Yang, J. , Summanen, P.H. , Henning, S.M. , Hsu, M. , Lam, H. , Huang, J. , *et al* (2015) Xylooligosaccharide supplementation alters gut bacteria in both healthy and prediabetic adults: a pilot study. Front Physiol 6: 216. 26300782 10.3389/fphys.2015.00216 PMC4528259

[mbt213514-bib-0057] Zasloff, M. (2002) Antimicrobial peptides of multicellular organisms. Nature 415: 389–395. 11807545 10.1038/415389a

[mbt213514-bib-0058] Zhang, B. , Lv, Z. , Li, H. , Guo, S. , Liu, D. , and Guo, Y. (2017) Dietary l‐arginine inhibits intestinal *Clostridium perfringens* colonisation and attenuates intestinal mucosal injury in broiler chickens. Br J Nutri 118: 321–332. 10.1017/S0007114517002094 28901890

[mbt213514-bib-0059] Zhou, Y.H. , Ma, H.X. , Yang, Y. , and Gu, W.M. (2018) Prevalence and antimicrobial resistance of Ureaplasma spp. and *Mycoplasma hominis* isolated from semen samples of infertile men in Shanghai, China from 2011 to 2016. Eur J Clin Microbiol Infect Dis 37: 729–734. 29313203 10.1007/s10096-017-3167-5

[mbt213514-bib-0060] Zhu, Y.G. , Johnson, T.A. , Su, J.Q. , Qiao, M. , Guo, G.X. , Stedtfeld, R.D. , *et al* (2013) Diverse and abundant antibiotic resistance genes in Chinese swine farms. Proc Natl Acad Sci USA 110: 3435–3440. 23401528 10.1073/pnas.1222743110 PMC3587239

[mbt213514-bib-0061] Zurfluh, K. , Stephan, R. , Widmer, A. , Poirel, L. , Nordmann, P. , Nuesch, H.J. , *et al* (2017) Screening for fecal carriage of MCR‐producing Enterobacteriaceae in healthy humans and primary care patients. Antimicrob Resist Infect Control 6: 28. 28316780 10.1186/s13756-017-0186-z PMC5351167

