# Supplementary figures and images for: Ethyl‐N‐dodecanoyl‐l‐arginate hydrochloride combats pathogens with low‐resistance generation by membrane attack and modifies gut microbiota structure
Source: Microb Biotechnol. 2019 Nov 22;13(3):722–37. doi: 10.1111/1751-7915.13514 (PMC7111106; doi:10.1111/1751-7915.13514)

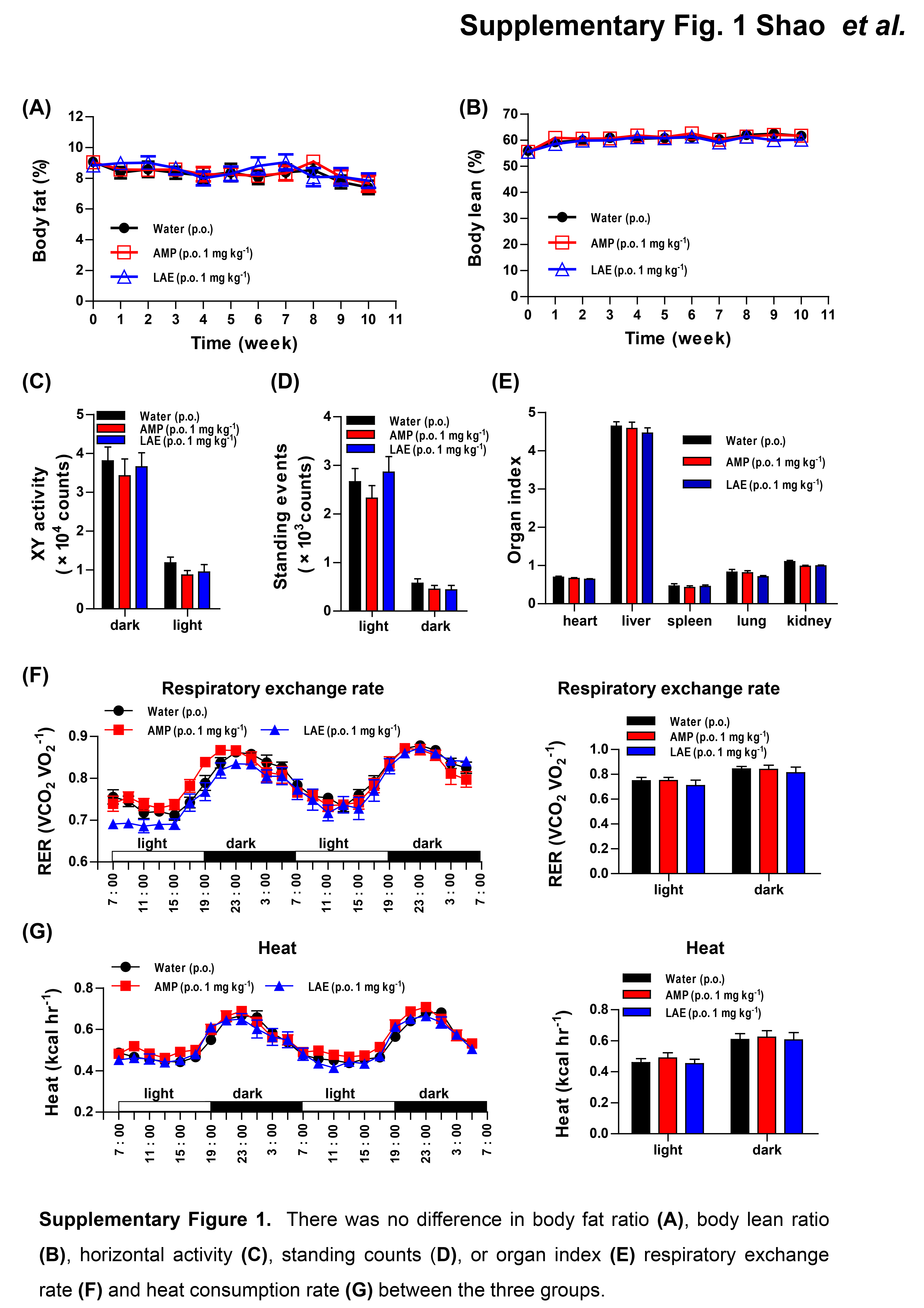

Supplement: Supplementary file 1 — Fig. S1. There was no different in body fat ratio (A), body lean ratio (B), horizontal activity (C), standing counts (D), or organ index (E), respiratory exchange rate (F) and heat consumption rate (G) between the three groups. [file MBT2-13-722-s001.tif]

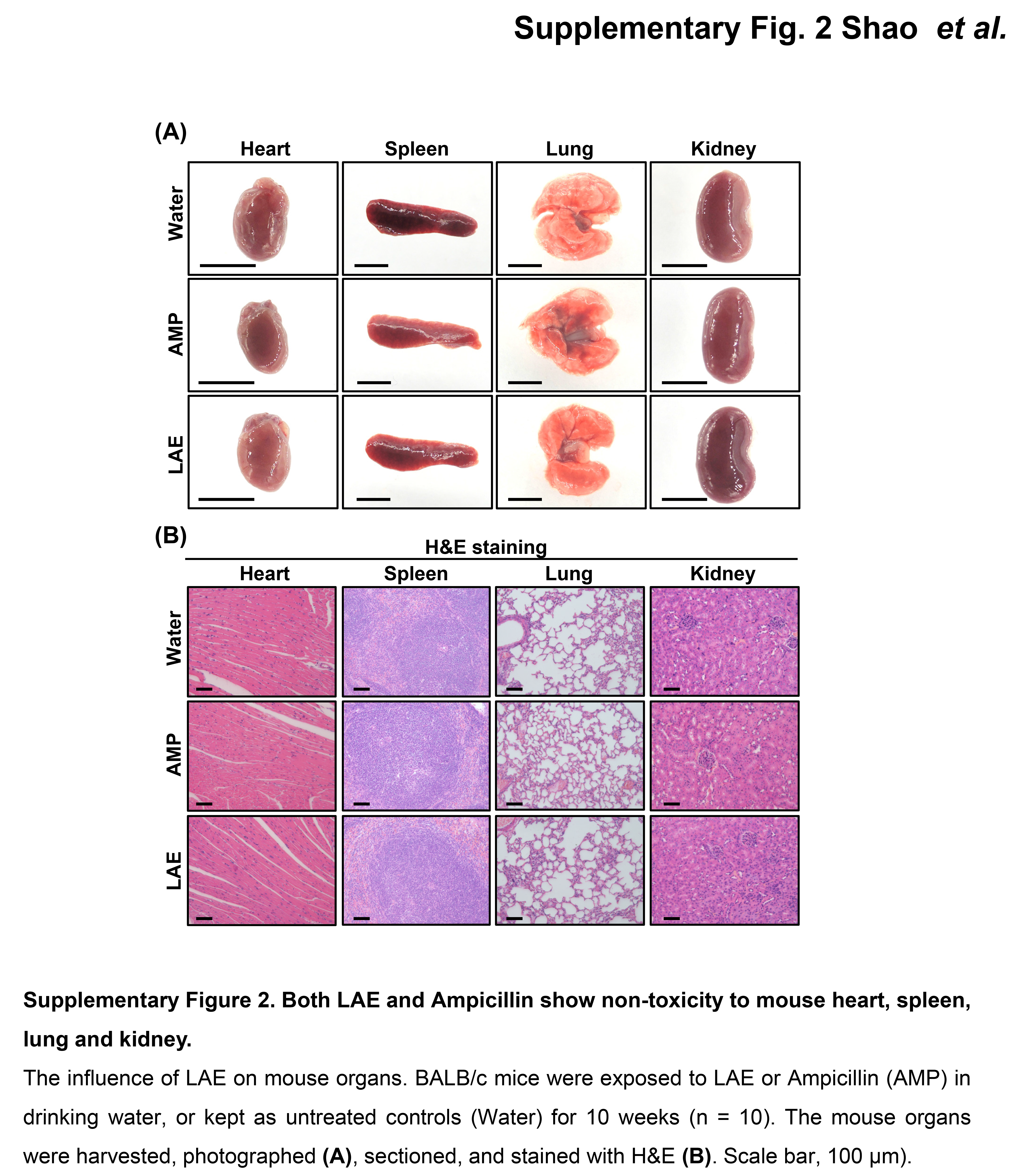

Supplement: Supplementary file 2 — Fig. S2. Both LAE and Ampicillin show non‐toxicity to mouse heart, spleen, lung and kidney. The influence of LAE on mouse organs. BALB/c mice were exposed to LAE or Ampicillin (AMP) in drinking water, or kept as untreated controls (Water) for 10 Weeks (n = 10). The mouse organs were harvested, photographed (A), sectioned, and stained, and stained with H&E (B). Scale bar, 100 μm. [file MBT2-13-722-s002.tif]

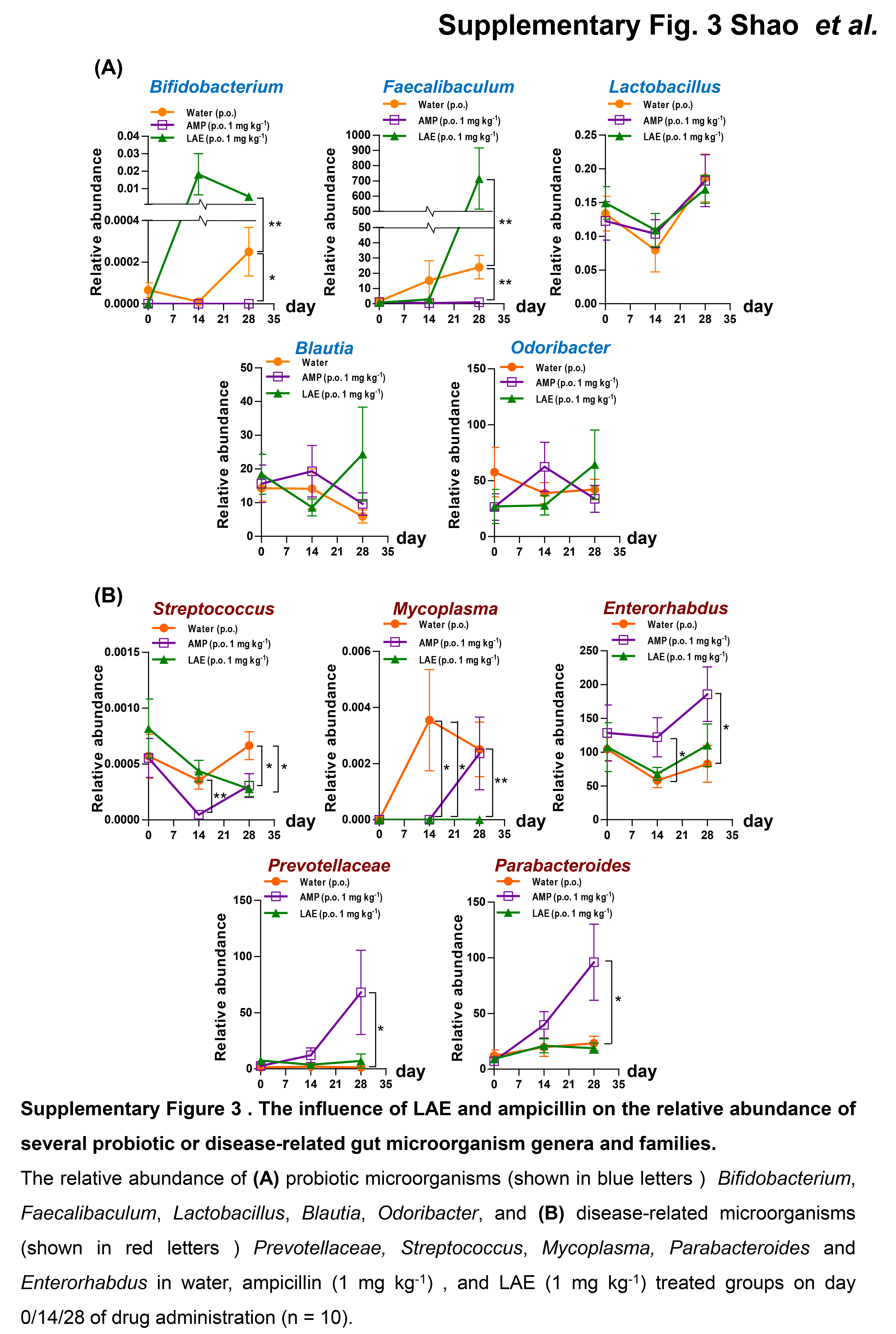

Supplement: Supplementary file 3 — Fig. S3. The influence of LAE and ampicillin on the relative abundance of several probiotic or disease‐related gut microorganism genera and families. The relative abundance of (A) probiotic microorganisms (shown in blue letters) Bifidobacterium, faecalibaculum, Lactobacillus, Blautia, Odoribactor, and (B) disease‐related microorganisms (shown in red letters) Prevotellaceae, Streptococcus, Mycoplama, paeabacteroids and Enterorhabsus in water, ampicillin (1 mg kg−1), and LAE (1 mg kg−1) treated groups on day 0/14/28 of drug administration (n = 10). [file MBT2-13-722-s003.tif]

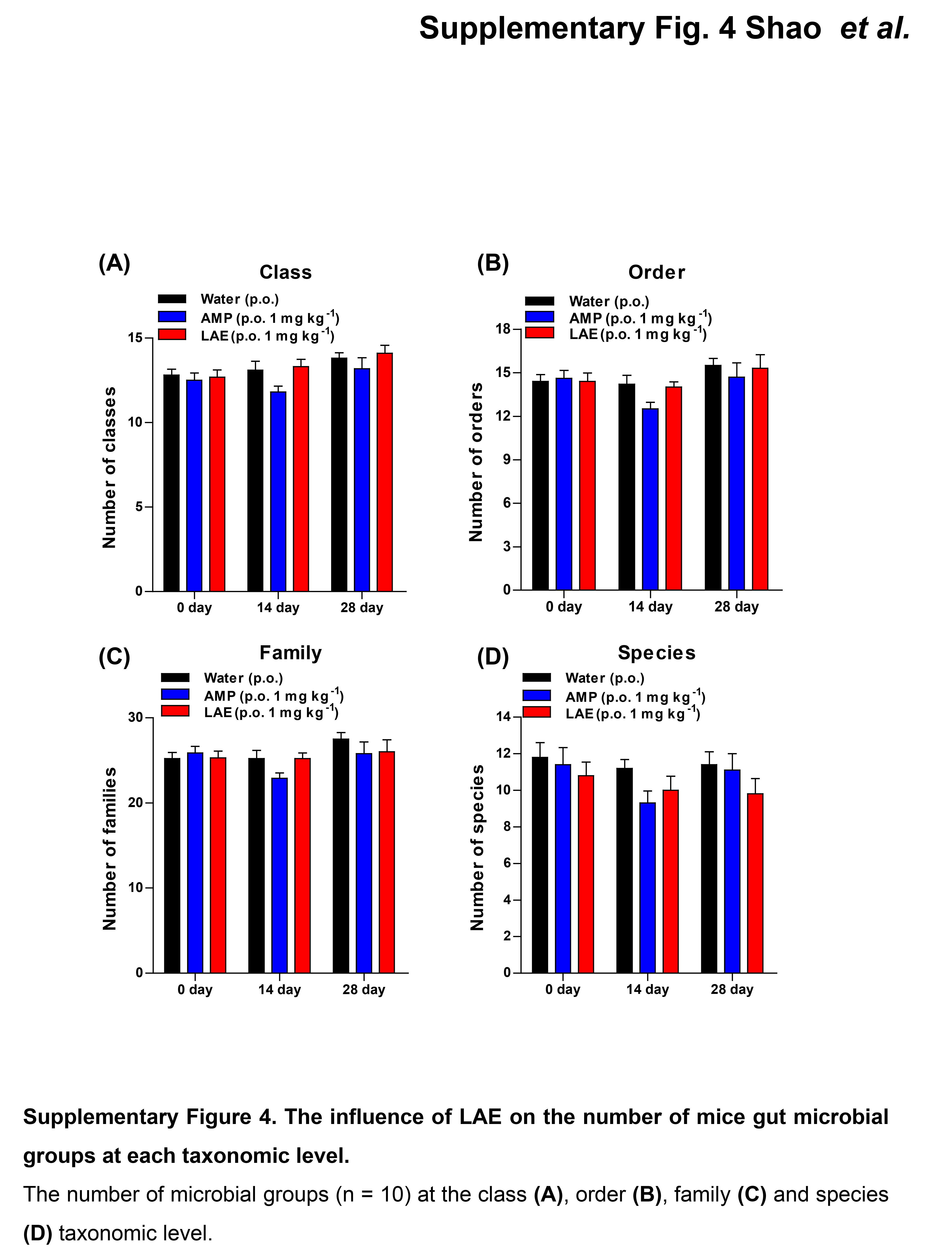

Supplement: Supplementary file 4 — Fig. S4. The influence of LAE on the number of mice gut microbial groups at each taxonomic level. The number of microbial groups (n = 10) at the class (A), order (B), family (C) and species (D) taxonomic level. [file MBT2-13-722-s004.tif]

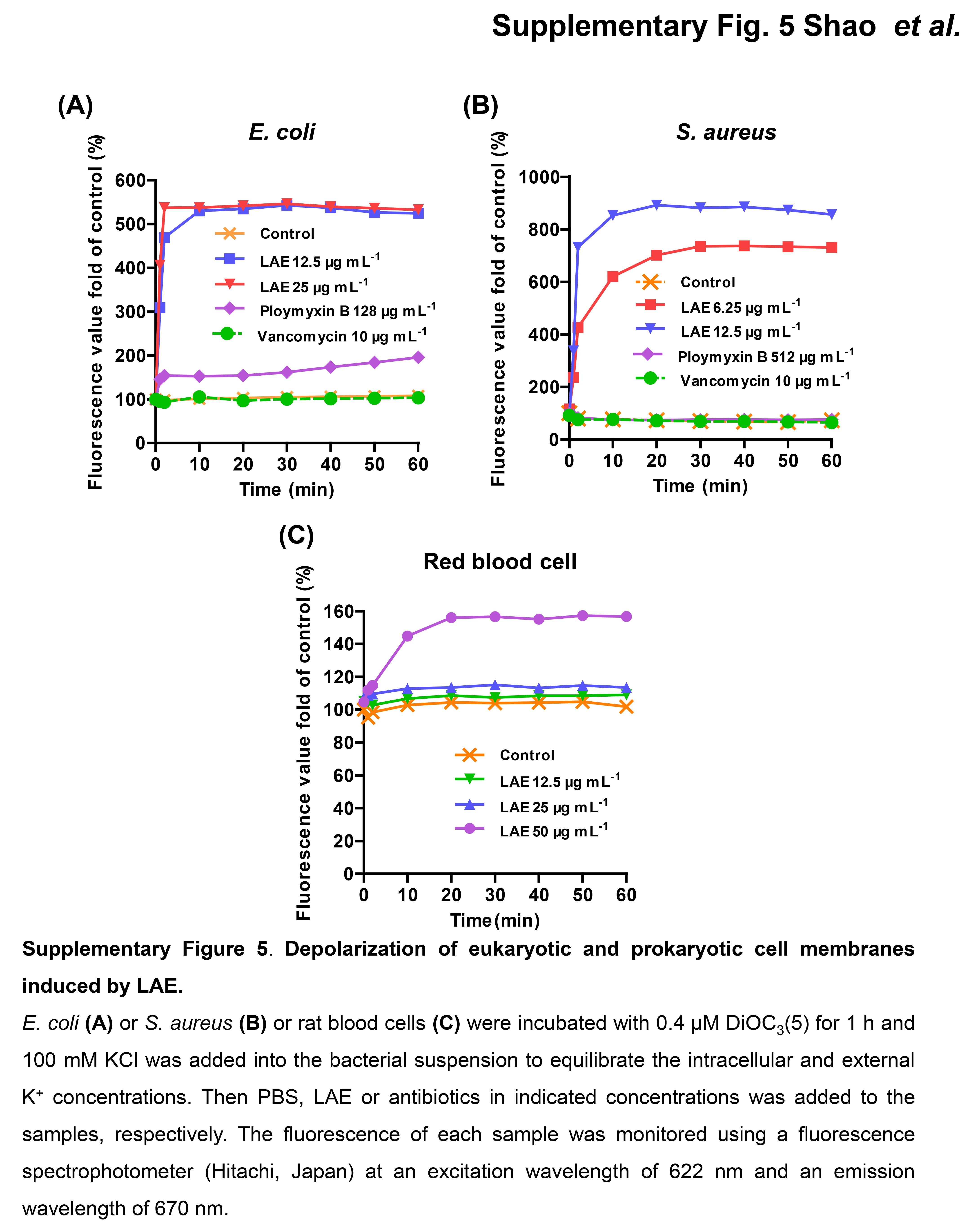

Supplement: Supplementary file 5 — Fig. S5. Depolarization of eukaryotic and prokaryotic cell membranes induced by LAE. E. coli (A) or S. aureus (B) or rat blood cells (C) were incubated with 0.4 μM DiOC3(5) for 1 h and 100 m M KCL was added into the bacterial suspension to equilibrate the intracellular and external K+ concentrations. Then PBS, LAE or antibiotics in indicated concentrations was added to the samples, respectively. The fluorescence of each sample was monitored using a fluorescence spectrophotometer (Hitachi, Japan) at an excitation wavelength of 622 nm and an emission wavelength of 670 nm. [file MBT2-13-722-s005.tif]
