# Supplementary material for: Ethyl‐N‐dodecanoyl‐l‐arginate hydrochloride combats pathogens with low‐resistance generation by membrane attack and modifies gut microbiota structure
Source: Microb Biotechnol. 2019 Nov 22;13(3):722–37. doi: 10.1111/1751-7915.13514 (PMC7111106; doi:10.1111/1751-7915.13514)
Supplement: Supplementary file 6 — Table S1. The effect of LAE on piglets yellow scour. [file MBT2-13-722-s006.docx]

| **Supplementray Table. 1The effect of LAE on piglets yellow scour** | | | | | | |
| --- | --- | --- | --- | --- | --- | --- |
| Groups | Drug doses  （mg kg^-1^ body weight） | The beginning number of piglets | Age  (day) | The final number of piglets | Survival rate of each age piglets | Survival rate  ±SD(%) |
| gentamicin | 50 mg kg^-1^ | 10 | 13 | 9 | 90.0% | 77.5%±11.9% |
|  |  | 12 | 12 | 8 | 66.7% |  |
|  |  | 8 | 11 | 7 | 87.5% |  |
|  |  | 10 | 9 | 7 | 70.0% |  |
| LAE | 10 mg kg^-1^ | 12 | 11 | 12 | 100.0% | 72.2%±45.1% |
|  |  | 7 | 2 | 5 | 71.4% |  |
|  |  | 10 | 9 | 9 | 90.0% |  |
|  |  | 7 | 3 | 0 | 0 |  |
|  | 50 mg kg^-1^ | 10 | 3 | 10 | 100.0% | 78.6%±23.5% |
|  |  | 11 | 11 | 9 | 81.8% |  |
|  |  | 12 | 12 | 10 | 83.3% |  |
|  |  | 9 | 13 | 4 | 44.4% |  |
